# Small effects of electric field on motor cortical excitability following anodal tDCS

**DOI:** 10.1016/j.isci.2024.108967

**Published:** 2024-02-01

**Authors:** Ilkka Laakso, Keisuke Tani, Jose Gomez-Tames, Akimasa Hirata, Satoshi Tanaka

**Affiliations:** 1 Department of Electrical Engineering and Automation, Aalto University, 02150 Espoo, Finland; 2 Faculty of Psychology, Otemon Gakuin University, Ibaraki, Osaka 567-8502, Japan; 3 Department of Medical Engineering, Graduate School of Engineering, Chiba University, Chiba 263-8522, Japan; 4 Department of Electrical and Mechanical Engineering, Nagoya Institute of Technology, Nagoya 466-8555, Japan; 5 Center of Biomedical Physics and Information Technology, Nagoya Institute of Technology, Nagoya 466-8555, Japan; 6 Laboratory of Psychology, Hamamatsu University School of Medicine, Hamamatsu, Shizuoka 431-3125, Japan

**Keywords:** Biological sciences, Neuroscience, Behavioral neuroscience, Techniques in neuroscience

## Abstract

The dose-response characteristics of transcranial direct current stimulation (tDCS) remain uncertain but may be related to variability in brain electric fields due to individual anatomical factors. Here, we investigated whether the electric fields influence the responses to motor cortical tDCS. In a randomized cross-over design, 21 participants underwent 10 min of anodal tDCS with 0.5, 1.0, 1.5, or 2.0 mA or sham. Compared to sham, all active conditions increased the size of motor evoked potentials (MEP) normalized to the pre-tDCS baseline, irrespective of anterior or posterior magnetic test stimuli. The electric field calculated in the motor cortex of each participant had a nonlinear effect on the normalized MEP size, but its effects were small compared to those of other participant-specific factors. The findings support the efficacy of anodal tDCS in enhancing the MEP size but do not demonstrate any benefits of personalized electric field modeling in explaining tDCS response variability.

## Highlights

• Anodal tDCS enhances cortical excitability, but electric fields show limited impact
• Other individual factors outweigh electric fields in anodal tDCS variability
• Personalized electric field modeling is unlikely to benefit the efficacy of tDCS

## Graphical abstract

**Figure:**
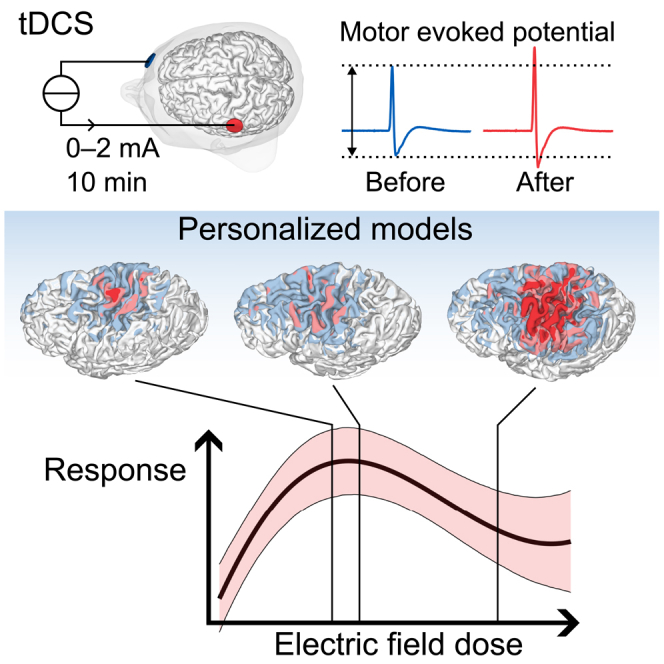

## Introduction

Applying a weak galvanic current through the intact scalp generates an electric field that polarizes the brain tissue, resulting in changes in the excitability of cortical neurons. This phenomenon makes transcranial direct current stimulation (tDCS) a potential therapeutic tool for various neuropsychiatric illnesses due to its capability to modulate neuroplasticity in humans.^1^ The effects of tDCS on neuroplasticity can be most easily investigated in the motor cortex, where it alters the size of motor evoked potentials (MEPs) measured using transcranial magnetic stimulation (TMS).^2^^,^^3^ These alterations include both acute effects during and immediately after stimulation and after-effects that can persist for hours or even longer after the stimulation.^4^

Physiological mechanism underlying tDCS still remain unclear. However, it has been reported that tDCS induces the changes in the membrane potential of the neurons under the electrodes, which alters the motor cortical excitability resulting in the modification of the MEP size.^3^^,^^5^ Previous studies have reported that the increases of the MEP sizes by tDCS were positively associated with the hand motor improvements in chronic stroke patients.^6^^,^^7^ Therefore, greater improvement in the MEP sizes may lead to more improvement in functional outcomes in some clinical settings.

However, the effects of stimulation parameters such as current and duration on inducing long-lasting changes in corticospinal excitability are not yet fully understood.^4^ Several studies have investigated the relationship between current and the MEP sizes within the range of typical tDCS currents (0.5–3 mA) and durations (7–20 min). For anodal tDCS, current-dependent differences that are possibly non-monotonic have been reported.^8^^,^^9^^,^^10^ Similarly, non-monotonic effects have also been reported for cathodal tDCS.^9^^,^^11^^,^^12^ In contrast, multiple studies have reported that anodal tDCS produces similar increases in excitability regardless of the stimulation current.^13^^,^^14^^,^^15^ Additionally, some studies have reported a lack of significant effects regardless of the stimulation current.^16^^,^^17^ Overall, despite the great variability between studies, it is evident that larger currents do not necessarily produce stronger aftereffects.^18^

The mixed results on dose response may be due to the fact that the prior investigators have measured the tDCS dosage in terms of the stimulation current, which is an indirect measure of the cortical electric field. Studies using computational current flow modeling have shown that these electric fields are influenced by the head anatomy, such as scalp, skull, and CSF thicknesses,^19^^,^^20^^,^^21^^,^^22^ leading to individual variability in the dosage when the stimulation current is fixed. Controlling the electric field dosage is possible with computational methods and might be the key to improve the reliability of tDCS.^23^ However, the relationship between the electric fields and the tDCS effects is still unclear. At present, only a few studies have experimentally investigated whether the variability in the electric fields is related to the variation in MEP sizes following motor-cortical tDCS, and these studies have reported partly contradictory results.^21^^,^^24^^,^^25^ For anodal tDCS with the M1–contralateral orbit montage, a positive dependence between electric field strength and excitability was found for 15 min of tDCS at 0.5–2 mA over the left hemisphere,^21^ a negative dependence for 20 min of tDCS at 1 mA over the right hemisphere,^24^ and no effect was observed for 10 min of tDCS at 2 mA over the left hemisphere.^25^ None of the studies have considered a nonlinear relationship between the electric field dosage and the MEP size modulation.

Here, our objective was to characterize the dose-response relationship of tDCS aftereffects by studying the changes in MEP sizes using a wide range of electric field strengths. We used the ‘fixed stimulator output–post hoc modeling’ paradigm,^18^ where each participant received a range of fixed currents (0, 0.5, 1.0, 1.5, and 2 mA) and the individual electric field in a location of interest was retrospectively calculated and correlated with MEP size modulation. To modify this approach,^18^ we used the anodal M1–contralateral orbit montage with 2 cm diameter electrodes that was previously found to produce up to 100% higher electric field strength in the targeted M1 than conventional large electrodes.^26^ The use of smaller electrodes is also expected to increase inter-individual variations in electric fields,^26^ which might lead to larger variations in MEP sizes and thus aid in the characterization of a potential dose-response relationship. Additionally, we investigated whether tDCS has differential effects on MEPs elicited by TMS test stimuli applied in the posterior-to-anterior direction (PA-TMS) and in the opposite direction (AP-TMS), which was previously observed for PA-tDCS and AP-tDCS electrode montages.^27^

Our specific research questions were: (Q1) does anodal tDCS with 0.5–2 mA currents change the MEP size compared to sham, (Q2) does tDCS change the MEP latency compared to sham, (Q3) do the MEP size changes measured using PA and AP test stimuli differ from each other, (Q4) what is the effect of individually calculated electric field on the MEP size, and (Q5) how large is the effect of the electric field on the MEP size modulation compared to other factors?

## Results

Data were collected from 21 participants who took part in 5 double-blind experimental sessions in counterbalanced order. MEP sizes and latencies were recorded from the first dorsal interosseous (FDI) muscle before tDCS and at 0, 15, and 30 min after tDCS. The MEP measurements were repeated for two directions of the TMS coil, inducing current in the PA or AP direction in the motor cortex.

To measure the MEPs, the test stimulus (TS) intensity used was 120% of the resting motor threshold (RMT). It is worth noting that the TS intensity might have slightly exceeded 120% RMT in some cases due to a mistake during the experiments (see STAR methods).

Magnetic resonance (MR) images of each participant were obtained prior to the tDCS experiments, to allow neuronavigated positioning of the TMS coil and the tDCS electrodes. After experiments, the MRI images were used to create individualized anatomical models that were used to model the electric fields generated in the brain by tDCS using the recorded electrode locations. The modeled electric field data could then be correlated with the MEP size modulations using linear mixed effect models.

### Side effects and tolerability

Stimulation was tolerated by all participants. Some participants reported mild discomfort such as tingling or slight pain in the scalp, which subsided within a few minutes after the stimulation started. No other side effects were observed.

### Baseline data

Average baseline MEP sizes and their standard deviations were 400±233 μV and 419±234 μV for PA- and AP-TMS, respectively. The corresponding TS intensities were 68±12% and 83±10% of the maximum stimulator output. Neither the baseline MEP sizes nor TS intensities differed significantly between sessions, which was tested using linear mixed effect models with either the TS intensity or the logarithm of the baseline MEP size as the dependent variable, condition as a fixed effect, and participant-specific intercepts as random effects. The TS intensity did not differ significantly between conditions for either PA- or AP-TMS ($\chi^{2}\left(4\right)=0.777$, p=0.9, and $\chi^{2}\left(4\right)=4.53$, p=0.3). The same was true for the logarithm of the baseline MEP size ($\chi^{2}\left(4\right)=3.1$, p=0.5, and $\chi^{2}\left(4\right)=0.623$, p=1).

### Anodal tDCS increases the normalized MEP size compared to sham

We first investigated whether active tDCS using 0.5–2 mA currents changed the normalized MEP size (MEP size divided by the baseline) compared to sham and whether there was a difference in the normalized MEP sizes for TS applied using PA- or AP-TMS.

A linear mixed effect model was used to analyze the dependence of the logarithm of the normalized MEP size on the stimulation current (0, 0.5, 1.0, 1.5, or 2 mA as a categorical variable) and TMS direction (PA or AP). The logarithm of the baseline MEP size, time point (0–30 min after tDCS), time of day (morning or afternoon), gender, their interactions with current, and session number were also included as predictors. The generalized extreme Studentized deviate test for the model residuals indicated one outlier data point that had a very small normalized MEP (participant #14, male, 1.5 mA, PA-TMS, 15 min after tDCS, normalized MEP: 0.17, baseline MEP: 480 μV) and was excluded from all analyses.

**Figure 1:**
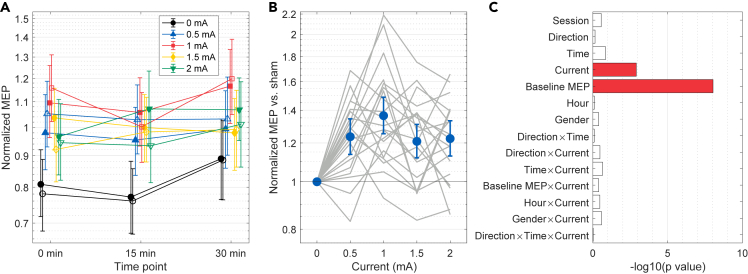
Partial dependence plots of the MEP size normalized to the baseline (A) Partial dependence of the normalized MEP size on current, time point, and TMS direction. Filled and empty markers indicate PA- and AP-TMS, respectively. Error bars are 95% confidence intervals obtained using bootstrapping. (B) Comparison of the active conditions to sham. Markers and error bars show the partial dependence of the ratio between the normalized MEP sizes of each condition and sham with 95% bootstrap confidence intervals. Gray curves show the partial dependencies calculated for each participant. (C) p-values of the fixed effects from likelihood ratio tests. Colored bars indicate statistically significant effects with false discovery rate of 0.05. See also Figure S1.

The fitted model is visualized in Figure 1A and its coefficients are summarized in Table S1. Likelihood ratio tests showed that the stimulation current ($\chi^{2}\left(4\right)=18.0$, p=0.001) and baseline MEP size ($\chi^{2}\left(1\right)=33.0$, $p=9\times 10^{-9}$) significantly affected the normalized MEP size. TMS direction did not have a significant effect on the normalized MEP size ($\chi^{2}\left(1\right)=0.142$, p=0.7). No significant effects were observed for other fixed effects (Figure 1C).

Inspection of the partial dependence plots (Figure 1A) and model coefficients (Table S1) indicated that active tDCS tended to produce larger normalized MEP sizes compared to sham. Partial dependence plot in Figure 1B compares the normalized MEP sizes of active conditions to those of sham, showing that all active tDCS conditions produced significantly larger normalized MEP size than sham. The increase was the largest for 1 mA current (+36%, 95% CI: +25–49%).

Alternative analysis using absolute instead of normalized MEP sizes indicated that the effects of current on the absolute MEP sizes were qualitatively similar to those observed for the normalized MEP sizes (Figure S1 and Table S3), sham stimulation tending to decrease the absolute MEP size and active conditions producing unchanged or slightly increased MEP sizes compared with the baseline (Figure S1).

### TDCS does not affect MEP latency, but time and TMS direction do

Next, we investigated whether tDCS changes the MEP latency differently compared to sham. We used a linear mixed effects model with the MEP latency difference as the dependent variable (see Table S5). The latency difference was calculated using the baseline time point of PA-TMS as the reference.

**Figure 2:**
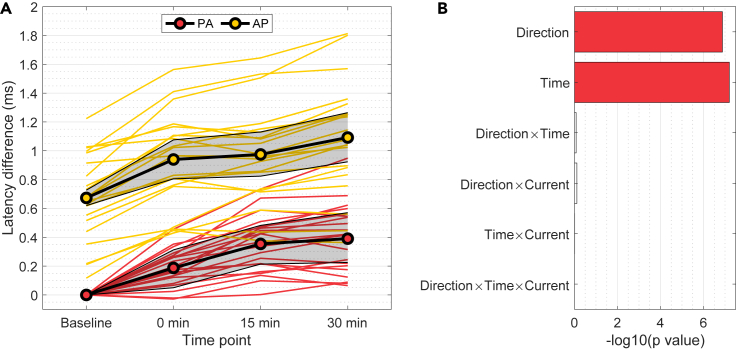
Dependence of the MEP latency on time point and TMS direction (A) Partial dependence plot of the MEP latency difference calculated using the baseline of PA-TMS as the reference. Shaded areas show the 95% bootstrap confidence intervals for the partial dependence. Colored curves show the partial dependencies for individual participants. (B) p-values of the fixed effects from likelihood ratio tests. Colored bars indicate statistically significant effects with false discovery rate of 0.05.

Partial dependencies calculated using the fitted model are illustrated in Figure 2A. Likelihood ratio tests showed no significant interaction effects of current (Figure 2B), indicating no differences between active and sham tDCS in the MEP latency. However, the MEP latency was significantly affected by the TMS direction ($\chi^{2}\left(1\right)=27.8$, $p=10^{-7}$) as well as time point ($\chi^{2}\left(3\right)=35.9$, $p=8\times 10^{-8}$). As illustrated in Figure 2A, AP-TMS MEPs had significantly longer latency than PA-TMS MEPs, the partial dependence of the difference being approximately 0.7 ms. The latencies of both PA- and AP-TMS MEPs increased with time in similar fashion, the average latency being approximately 0.4 ms longer at 30 min after stimulation than at the baseline.

### Electric field has a nonlinear effect on the MEP size

**Figure 3:**
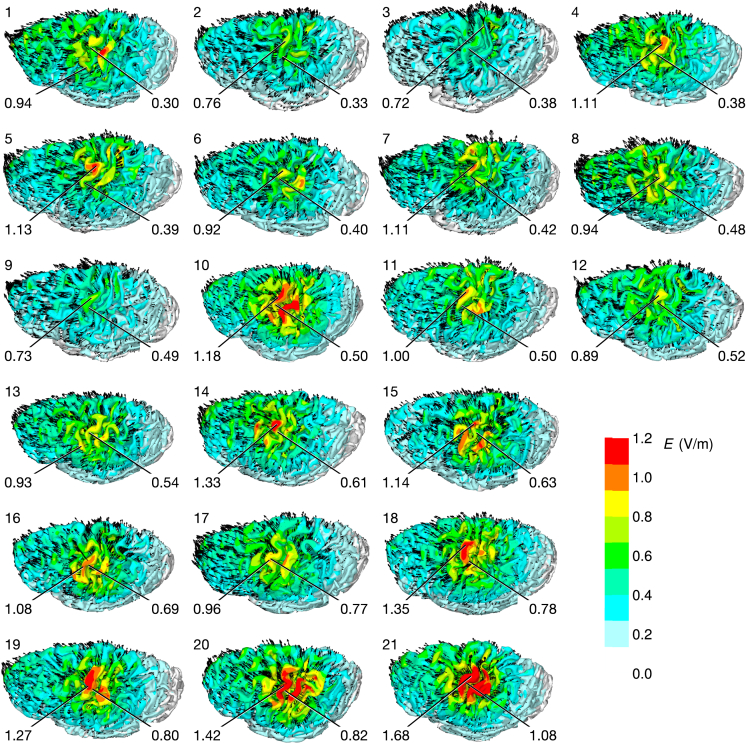
Calculated electric field in each participant for 1 mA current Panels labeled 1–21 show the electric field strength for each participant at a depth of 2 mm below the surface of the gray matter. Arrows indicate the direction of the field. The left (blue) and right (red) labels indicate the maximum electric field strength over the precentral gyrus and the $E_{\text{FDI}}$ values and their locations, respectively. The participants are sorted in the ascending order by the $E_{\text{FDI}}$ value.

We modeled the electric field in the brain of each participant using the finite-element method in MRI-based models. Figure 3 visualizes the calculated electric fields.

Our previous study suggested that there was a relationship between the MEP size and the normal component of the electric field calculated at cortical site activated by TMS.^24^ Therefore, FreeSurfer was used to map [−41,−7,63], corresponding to the estimated activation site for the FDI muscle,^28^ from the standard brain space to the brain of each participant. The normal component of the electric field at this location was then calculated and is denoted by $E_{\text{FDI}}$ in the following. For 1 mA current, the summary statistics of $E_{\text{FDI}}$ were: mean ± SD: 0.56±0.20 V/m and range: 0.29–1.08 V/m.

**Figure 4:**
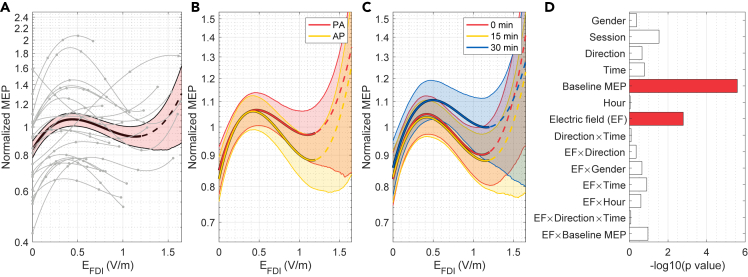
Effect of the electric field normal component on the normalized MEP size (A) Partial dependence of the normalized MEP for the electric field normal component (black curve). Shaded area indicates the 95% bootstrap confidence bounds. The gray curves show the partial dependence for individual participants. The observed $E_{\text{FDI}}$ values in each participant are marked with gray dots. (B and C) Partial dependence of the normalized MEP size on $E_{\text{FDI}}$ for PA- and AP-TMS (B) and for each time point (C). Shading indicates 95% confidence bounds. (D) p-values of likelihood ratio tests. Colored bars indicate statistically significant effects with false discovery rate of 0.05. See also Figure S2.

To investigate how the normalized MEP size depends on individually calculated $E_{\text{FDI}}$ values, we modeled the effect of the electric field using a linear mixed effect model (see Table S2). To allow a nonlinear relationship, first, second and third-order orthonormal polynomials of $E_{\text{FDI}}$ and their interactions with other experimental factors (listed in Figure 4D) were included as predictors. Likelihood ratio tests indicated a significant effect of the electric field on the normalized MEP size ($\chi^{2}\left(3\right)=15.2$, p=0.002). Further investigation using likelihood ratio tests showed that the third-order term was statistically significant ($\chi^{2}\left(9\right)=13.5$, p=0.003), indicating that the effect of the electric field was nonlinear. Lower-order terms were not significant (p>0.1).

The effect of the electric field on the normalized MEP size is visualized in Figures 4A–4C. There were relatively large inter-participant differences (Figure 4A) and no significant effects of TMS direction (Figure 4B) or time point (Figure 4C) on the relationship between $E_{\text{FDI}}$ and the normalized MEP size. On average, the normalized MEP size was the smallest when $E_{\text{FDI}}$ was 0 V/m, which corresponds to sham. The largest normalized MEP size was reached for $E_{\text{FDI}}$ of 0.44 V/m (95% CI: 0.38–0.55 V/m), after which the normalized MEP size decreased. The difference in the normalized MEP size between 0 V/m and the maximum was +27% (95% CI: 17–37%). The effects of $E_{\text{FDI}}$ on the absolute MEP sizes are visualized in Figure S2. At the baseline time point, there was no effect of $E_{\text{FDI}}$ on the absolute MEP size, and, at post-tDCS time points, the dependence of the absolute MEP size on $E_{\text{FDI}}$ had a similar nonlinear tendency to that observed for the normalized MEP sizes (Figure S2).

### The effect of the electric field is small compared to participant-specific factors

**Figure 5:**
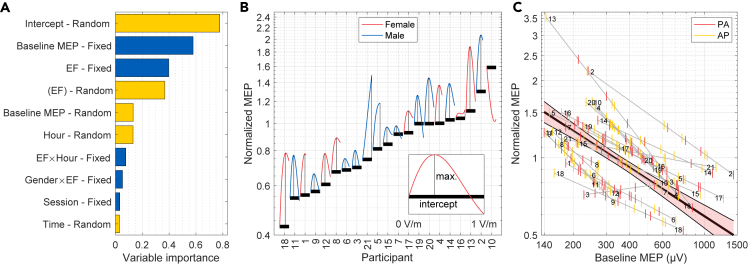
Effects of the most important non-electric field factors on the normalized MEP size (A) Variable importance (arbitrary units) of various predictors on the coefficient of determination obtained using the permutation method. Ten predictors with the largest variable importance are shown. The importance of fixed and random effect predictors are indicated by blue and yellow bars, respectively. The fourth most important predictor is written in parentheses as it includes the combined effects of both electric field and Session (see text). (B) Partial dependence plot of the participant-specific intercepts and the estimated participant-specific effect of $E_{\text{FDI}}$ in the range of 0–1 V/m. The participants are sorted in the ascending order by the intercept. (C) Partial dependence of the normalized MEP on the baseline MEP size. Overall partial dependence and its 95% confidence bounds are indicated by the thick black line and the shaded area, respectively. Gray line segments show the partial dependence for each participant. Vertical line segments indicate the measured values of the baseline MEP for each participant for PA- and AP-TMS. Participant numbers are shown in both ends of the line segments, and they correspond to those in B and in Figure 3.

To study which factors have the largest effect on the normalized MEP size, we estimated the variable importance of both fixed and random effect predictors using the permutation method. The estimated variable importance of each factor is illustrated in Figure 5A. There were three predictors whose variable importances were greater than or similar to that of the electric field.

The participant-specific intercept had the largest variable importance. Figure 5B illustrates that the effect was a ‘level effect’, wherein each participant tended to respond similarly to each intervention, regardless of the electric field. For instance, the MEP sizes decreased for all $E_{\text{FDI}}$ values in some participants (left side of Figure 5B), but for other participants, the MEP size increased for all conditions (right side of Figure 5B).

The second most important predictor was the fixed effect of baseline MEP size. There was a negative dependence between the normalized MEP size and the baseline MEP size (Figure 5C), larger baseline MEPs leading to smaller normalized MEP sizes. This effect is not a real physiological effect, but rather a technical consequence of normalization that is well known for TMS protocols that use normalized MEP as the dependent variable.^29^^,^^30^ A large/small initial measurement is more likely to decrease/increase for a second measurement, leading to a negative slope between the normalized MEP size and the baseline MEP size.^29^^,^^30^

The fourth most important predictor term was the participant-specific effect of $E_{\text{FDI}}$, which modeled the differences in the participants’ responses to the electric field and is illustrated in Figure 5B. The predicted individual optimal $E_{\text{FDI}}$ values differed between participants and their summary statistics were: mean ± SD: 0.61±0.44 V/m and range: 0–1.54 V/m. These $E_{\text{FDI}}$ values corresponded to 0–138% (mean ± SD: +39±30%, median: +35%) larger normalized MEP sizes than sham, depending on the participant. However, the participant-specific effect of $E_{\text{FDI}}$ was likely overestimated by our model because the predictor term also included the variability due to participant-specific inter-session variations. The reason is that each $E_{\text{FDI}}$ value was used in only a single experimental session, and thus, the effects of session and $E_{\text{FDI}}$ could not be separated at the participant level. Nevertheless, these inter-participant differences in the effects of session and/or $E_{\text{FDI}}$, which cannot be easily controlled, had comparable variable importance to the fixed effect of $E_{\text{FDI}}$.

Other predictor terms had minor importance and were not statistically significant.

### Method for calculating the electric field has little effect on the prediction

Finally, we studied the sensitivity of the model to the choice of the electric field outcome measure and model parameters. To study the effects of model parameters on the predicted effect of the electric field, we changed the segmentation method for generating the head models from MRI data, and decreased/increased the electrical conductivity values of the cerebrospinal fluid (CSF) and bone, which are the tissues/bodily fluids with the highest and lowest conductivities, respectively. To characterize the effect of the electric field outcome measure, three additional measures were considered: the strength of the electric field at [−41,−7,63] (${\left|E\right|}_{\text{FDI}}$), the average electric field strength over a spherical region of interest with 3 cm diameter ($E_{\text{sphere}}$), and the average electric field in a region of interest where the TMS-induced electric field was greater than 70% of its maximum ($E_{\text{TMS}}$). Furthermore, we tested the effect of changing the location of interest from [−41,−7,63] to various other locations in the cerebral cortex.

The linear mixed effects models were refitted for each combination of model parameters and outcome measure, and the electric value ($E_{\text{opt}}$) producing the largest increase in the normalized MEP size compared with sham (Δ NMEP) was determined. The results of this sensitivity analysis are listed in Table 1 and the effects of changing the location of interest are illustrated in Figure S3.

**Table 1:** Sensitivity of the findings to the parameters used for the electric field modeling

Model	Measure	Mean ± SD	$E_{\text{opt}}$	Δ NMEP
1 (default)	$E_{\text{FDI}}$	0.56±0.20	0.44	+27%
	${\left|E\right|}_{\text{FDI}}$	0.74±0.20	0.60	+28%
	$E_{\text{sphere}}$	0.64±0.14	0.49	+31%
	$E_{\text{TMS}}$	0.86±0.18	0.67	+29%
2 (alternative segmentation)	$E_{\text{FDI}}$	0.54±0.19 (N.S.)	0.55	+24%
	${\left|E\right|}_{\text{FDI}}$	0.70±0.22 (N.S.)	0.68	+25%
	$E_{\text{sphere}}$	0.59±0.19 (N.S.)	0.56	+26%
	$E_{\text{TMS}}$	0.77±0.22∗	0.70	+25%
3 ($\sigma_{\text{CSF}}$: −10%)	$E_{\text{FDI}}$	0.59±0.21∗∗∗	0.46	+27%
	${\left|E\right|}_{\text{FDI}}$	0.78±0.20∗∗∗	0.62	+28%
	$E_{\text{sphere}}$	0.67±0.14∗∗∗	0.52	+31%
	$E_{\text{TMS}}$	0.91±0.18∗∗∗	0.71	+29%
4 ($\sigma_{\text{CSF}}$: +10%)	$E_{\text{FDI}}$	0.53±0.19∗∗∗	0.42	+27%
	${\left|E\right|}_{\text{FDI}}$	0.70±0.19∗∗∗	0.57	+28%
	$E_{\text{sphere}}$	0.61±0.13∗∗∗	0.47	+31%
	$E_{\text{TMS}}$	0.82±0.18∗∗∗	0.64	+29%
5 ($\sigma_{\text{bone}}$: −50%)	$E_{\text{FDI}}$	0.45±0.14∗∗∗	0.35	+27%
	${\left|E\right|}_{\text{FDI}}$	0.58±0.14∗∗∗	0.46	+28%
	$E_{\text{sphere}}$	0.51±0.11∗∗∗	0.39	+31%
	$E_{\text{TMS}}$	0.66±0.14∗∗∗	0.51	+29%
6 ($\sigma_{\text{bone}}$: +50%)	$E_{\text{FDI}}$	0.62±0.23∗∗∗	0.49	+27%
	${\left|E\right|}_{\text{FDI}}$	0.83±0.23∗∗∗	0.68	+28%
	$E_{\text{sphere}}$	0.70±0.16∗∗∗	0.55	+30%
	$E_{\text{TMS}}$	0.97±0.20∗∗∗	0.77	+28%

Models 1–6 correspond to different choices of model parameters and measures correspond to different outcome measures (see text). The mean and standard deviation of each electric field measure are reported for 1 mA current. The means are compared to those of Model 1 using paired Student’s t-tests (N.S.: p>0.05, ∗: p<0.05, ∗∗: p<0.01, ∗∗∗: p<0.001). $E_{\text{opt}}$ is the electric field value that maximizes the normalized MEP size and Δ NMEP is the change in the normalized MEP size compared with the sham condition for $E_{\text{opt}}$. See also Figure S3.

The results in Table 1 and Figure S3 showed that the choice of model parameters, outcome measures, and locations of interest affected both the mean electric field and $E_{\text{opt}}$ values. The conductivities of CSF and bone had small systematic negative and positive effects, respectively, on the electric field values, but the segmentation method did not have a significant effect. However, the effects of model parameters and outcome measure on Δ NMEP were minor. For instance, none of the alternative model parameters or outcome measures listed in Table 1 increased Δ NMEP by more than 4 percentage points. Therefore, the relatively weak effect of the electric field on the normalized MEP size was not due to a suboptimal choice of the model parameters or the outcome measure.

## Discussion

Based on the analysis of the experimental data, the answers to our specific research questions were: (Q1) on average, active tDCS conditions with 0.5–2 mA current increased the normalized MEP size compared to sham (Q2), tDCS did not affect the MEP latency differently from sham, (Q3) the effect of active tDCS or sham on the MEP size did not depend on the direction of TMS test stimuli, (Q4) the individually-modelled electric field had a nonlinear effect on the normalized MEP size, but (Q5) the effects of electric field were weak compared to those of other factors.

### Limited value of electric field modeling in predicting MEP sizes

The results revealed statistically significant effects of both the stimulation current and electric field on the normalized MEP size. When using the stimulation current as the metric for tDCS dosage, a conventional approach in tDCS studies, the largest increases in the normalized MEP size compared to the sham condition were produced by a 1 mA current (+36% increase from sham). This increase aligns with findings from previous studies using currents in the same range,^9^^,^^15^ despite discrepancies in experimental details such as duration, electrode size, TS intensity, and TMS current direction. Moreover, differences between the active conditions were minor, consistent with previous research as well.^9^^,^^13^^,^^14^^,^^15^

When assessing the tDCS dosage using the individual $E_{\text{FDI}}$ value, the statistical model indicated a nonlinear dose-response relationship characterized by a peak normalized MEP increase at 0.44 V/m. This ‘optimal’ $E_{\text{FDI}}$ value led to a +27% increase in the normalized MEP size compared to the sham condition. The nonlinear nature of the effect resulted in certain ranges where the influence of $E_{\text{FDI}}$ on the normalized MEP size could be either positive, negative, or negligible. This variability might explain the mixed outcomes encountered in prior tDCS studies that combined experiments with electric field modeling,^21^^,^^24^^,^^25^ yielding positive, no, and negative effects, respectively. Nevertheless, a direct comparison of these findings to our study is unfeasible due to discrepancies in experimental parameters. The nonlinear effect may be explained by a model wherein the electric field modulates the polarization of axon terminals of cortical neurons in a nonlinear fashion.^31^

Despite the significant effect of $E_{\text{FDI}}$, the results do not support the hypothesis that the efficacy of tDCS protocols can be enhanced through personalized dosing guided by electric field modeling.^23^^,^^32^^,^^33^ If $E_{\text{FDI}}$ were a superior dosage measure compared to the stimulation current, we would anticipate a significantly larger predicted response when each participant’s $E_{\text{FDI}}$ was fixed to an optimal value (0.44 V/m) than when their current was fixed to its optimal value (1 mA). However, this was clearly not the case; the predicted effect of the fixed $E_{\text{FDI}}$ was, on average, a smaller increase in the normalized MEP size compared to the sham condition than that observed with a fixed current. Additionally, the linear mixed effect models indicated that the effect of $E_{\text{FDI}}$ was participant-specific, suggesting that the optimal $E_{\text{FDI}}$, if it exists, varies between participants. This observation negates much of the potential benefits of personalized tDCS dosing based on electric fields, given that the optimal electric field dose for each new subject will be unknown. These inter-individual variations may be caused, e.g., by the angle between the electric field and directions of the affected cortical neurons,^31^ which would be difficult to measure in the intact brain.

If the electric field in the brain is the physical agent of tDCS, then why did its impact appear limited compared with that of the stimulation current alone? A likely explanation was that the inter-individual variability in the sensitivity, discussed above, masked the effects at the group level. Another potential explanation could be that the dose-response characteristics are so complex^18^ that they could not be satisfactorily represented using the present model. We tested whether the effect of the electric field could be strengthened by using various electric field outcome measures and model parameters, but none of the tested measures/parameters provided a substantially different fit between the electric field and the normalized MEP size. Nevertheless, it is still possible that the electric field can have a larger effect than that reported in our study, but it remains an open question how the electric field data should be processed to obtain key values having good predictive power for tDCS responses.^34^

In future studies of dose-response characteristics, it would be advantageous to focus on the range of 0–0.3 V/m, where the predicted effect of the electric field was strongest (displaying the steepest derivative). Employing multiple electrode configurations, even ones that ‘miss’ the intended M1 target, could help identify suitable location(s) of interest and confirm whether the electric field is a better dosage measure than current. Finally, we note that our models have been fitted in the range of 0–2 mA and cannot be used for extrapolation to larger currents/electric fields, e.g., to 4 mA used in some recent studies.^35^^,^^36^

### No effect of TMS coil direction on MEP size modulation

The modulation of the normalized MEP size due to tDCS was insensitive to the orientation of the TMS coil, tDCS facilitating the MEPs of both PA-TMS and AP-TMS in the same way. Despite no differences in the MEP size modulation, we found a consistent, approximately 0.7 ms, latency difference between the PA- and AP-TMS MEPs measured in resting muscles.

For suprathreshold stimuli in active muscles, the PA–AP latency difference is already visible at the cervical level in the spinal cord, where suprathreshold AP-TMS generated the same I-waves as PA-TMS but with a 0.2–0.7 ms longer latency.^37^ The latency difference pointed to the activation of two different cortical circuits by PA- and AP-TMS,^37^ perhaps different inputs to the same or different corticospinal tract cells.^38^ Hence, it is likely that the latency difference observed herein using suprathreshold TMS in resting muscles was also due to activation of different structures at the cortical level, although it is still unclear what these activated structures were.^39^

The nearly identical effects of PA- and AP-TMS imply that the outputs of both pathways were comparably influenced by tDCS. This suggests that the effect of tDCS was not selective at the cortical level. Previous findings have indicated distinct effects of 1 mA tDCS on PA- and AP-TMS MEPs when utilizing a bipolar electrode configuration.^27^ However, the bipolar electrode setup might target different subregions of M1 compared to the unipolar montage used in our study,^26^^,^^40^ which may explain the absence of difference between PA- and AP-TMS responses.

### Delayed MEP latency indicates changes in body temperature

We found that the time point after stimulation significantly affected the MEP latency, later time points producing approximately 0.4 ms (at 30 min after stimulation) longer latency than at the baseline. The latency delay was similar for all active conditions and sham. The effect was remarkably consistent between participants and experimental sessions.

The most likely explanation for the delay in latency is that, because the participants were sitting still, the temperature of the arms slightly decreased due to physical inactivity. The decrease in the temperature slowed down the conduction velocity in peripheral nerves, leading to a longer latency. The relative increase in the latency was approximately 1.6–2%, which corresponds to a 0.3°C–0.4°C decrease in temperature^41^ and is well within the normal physiological range.

Despite the robustness of the effect, we are not aware of previous studies reporting MEP latency changes following tDCS. It was previously shown that local cooling of the scalp by 12.5°C during 10 min had no effect on the MEP latency.^42^ However, such local cooling is unlikely to affect the nerve conduction velocity at the periphery.

If the decrease in the temperature also occurred in the brain, it could have led to a decreased MEP size that was observed following sham stimulation. Namely, local cooling of the scalp has been shown to depress corticomotor excitability,^42^ although such local cooling did not affect the MEP latency.^42^ We also note that body temperature is correlated with cognitive performance and alertness,^43^ which could also be related to the reduction of the MEP size following the sham condition.

To confirm that the latency change is not specific to some undocumented experimental factor such as room temperature, we invite others to report MEP latencies and/or body temperature changes in future studies. If the body temperature changes during the experiment, it may need to be considered in the experimental design.

### Individuals tend to respond similarly regardless of condition

The variability of the participant-specific intercepts was the largest variance component and resulted in some participants consistently decreasing or increasing the normalized MEP size, regardless of the stimulation current or electric field. This participant-specific baseline effect was not caused by the order of the experimental sessions, sex, variations in the baseline MEP size, or time of day effects,^44^ as these were controlled using a linear mixed effects model. Additionally, this effect was not explicable by possible skin sensations or issues related to blinding (see ‘limitations of the study’), as it was consistent across all active conditions and the sham condition.

Potential differences in individual attentional levels during tDCS might offer an explanation, as the tDCS effect on the MEP size is influenced by the attention to the target hand.^45^ We controlled the participants’ attention level by asking them to direct their attention to the right FDI muscle. However, the attention level might have varied more among participants than among experimental sessions. This difference could have led to inter-individual trends of the tDCS effect including the sham condition. The relatively high consistency between sessions within participants could also be linked to robust intra-individual reliability of anodal tDCS, participants tending to respond similarly to multiple applications of the same condition.^9^^,^^46^

The large baseline effect indicates that the normalized MEP size alone is a poor measure of an individual’s response to tDCS. For instance, a normalized MEP size smaller than unity might actually mean that tDCS had a facilitatory effect on the MEP size, depending on the individual’s baseline level. If the baseline effect is not specific to the experimental conditions of this study, future investigations of individual tDCS dose-response should feature multiple experimental sessions, including the sham condition, so that the participants’ baseline levels can be accurately characterized.

### Limitations of the study

There were two main limitations. First, following an experimental oversight, the TS intensity might have slightly exceeded the planned intensity of 120% RMT depending on the experimental session and participant, which could have led to more variability in the MEP sizes. However, the effect appeared to be minor. Variability between participants in the MEP size had a coefficient of variation (CV) of 57%, which was comparable to the average CV of 52% in TMS studies using TS intensity of 120% RMT.^30^ Additionally, larger than planned TS intensity could have led to atypical MEP sizes. This did not seem to be case either. At the baseline, the mean and standard deviations of the MEP sizes were 409±234 μV. Typically, the average MEP size for 120% RMT is 870 μV, and the average MEP sizes vary between studies with a CV of 29%.^30^ Given these large inter-study variations, the MEP sizes in this study are comparable to those obtained using 120% RMT. They fall into the low end of the normal MEP size range, but are not unexpectedly small (−1.8 SDs from the average). Therefore, our findings should be comparable to studies that have used TS intensity of 120% RMT.

The second main limitation was that we did not collect quantitative data on skin sensation or reliability of blinding. Previous data on scalp sensations does not currently exist for 2 cm diameter electrodes in the M1–contralateral orbit montage. However, data from larger electrode sizes (16 cm^2^ vs. 35 cm^2^) suggested that smaller electrodes do not lead to stronger skin sensations when the current is fixed in the range 0.5–1.5 mA.^47^ Additionally, multiple studies on high-definition (HD) tDCS have used electrode sizes similar to or smaller than those used in our study and have reported no quantitative differences in scalp sensations compared to conventional tDCS for 2 mA^48^^,^^49^ and 1 mA^50^ currents. Therefore, we expected no major differences in scalp sensations compared to earlier studies, but effects due to skin sensations cannot be fully excluded.

Finally, we note that the TMS coil placement was done based on anatomical images only, necessitated by the use of both PA- and AP-TMS, whereas the coil is more typically positioned at the functional hotspot in tDCS studies. This difference may need to be considered whenever our results are compared to previous or future data. A different coil location might have resulted in a gentler slope of the MEP input–output curve, or a lower ceiling for the MEP size, and may have contributed to the smaller than average MEP sizes, mentioned above.

## STAR★Methods

### Key resources table

**Table:** 

REAGENT or RESOURCE	SOURCE	IDENTIFIER
**Deposited data**		

Data for ‘Small effects of electric field on motor cortical excitability changes following anodal transcranial direct current stimulation’	This paper; Mendeley Data	https://doi.org/10.17632/y2nr4gzsjt.1

**Software and algorithms**		

MATLAB version R2022a	The MathWorks, Inc.	https://www.mathworks.com
Voxel-based finite-element method solver with geometric multigrid	Laakso and Hirata^51^	https://version.aalto.fi/gitlab/ilaakso/vgm-fem
FreeSurfer version 7.1.1	Dale et al.^52^; Fischl et al.^53^^,^^54^	https://surfer.nmr.mgh.harvard.edu

### Resource availability

#### Lead contact

Further information and requests for resources should be directed to and will be fulfilled by the lead contact, Ilkka Laakso (ilkka.laakso@aalto.fi).

#### Materials availability

This study did not generate new unique reagents.

#### Data and code availability

• Measured and calculated data have been deposited at Mendeley Data and are publicly available as of the date of publication. DOIs are listed in the key resources table.
• This paper does not report original code.
• Any additional information required to reanalyze the data reported in this paper is available from the lead contact upon request.

### Experimental model and study participant details

Twenty-one (12 males, 9 females, aged 21–28 years) healthy right-handed volunteers participated in this study. All participants were medical or nursing students of Japanese descent, were naive to the purpose of this study, and had no neurological and cognitive disorders. After a full explanation of the experimental procedures, written informed consent was obtained from all participants. This study was approved by a local ethics committee of Hamamatsu University School of Medicine and was conducted in accordance with the Declaration of Helsinki.

### Method details

#### Imaging

Prior to the experiments, all participants underwent structural magnetic resonance imaging (MRI). T1 and T2 weighted MR images for each participant were obtained using a 3.0 T MRI scanner (Discovery MR750 3.0 T, GE Healthcare Japan, Japan). The scan parameters for T1 were as follows: repetition time (TR)/echo time (TE)/flip angle (FA)/field of view (FOV)/voxel size = 7.2 ms/2.1 ms/15°/256 mm × 256 mm/1 mm × 1 mm × 1 mm T2-weighted image was acquired with the following parameters: TR/TE/FA/FOV/voxel size = 2500 ms/75.5 ms/90°/256 mm × 256 mm/1 mm × 1 mm × 1 mm.

#### tDCS

The present study was designed as double-blind and sham-controlled to assess the current-dependent effect of anodal tDCS on the corticospinal excitability.

Anodal tDCS was applied for each participant using a DC STIMULATOR PLUS (neuroConn GmbH, Ilmenau, Germany) with five conditions (sham, 0.5, 1.0, 1.5, and 2.0 mA). Anode and cathode electrodes (neuroConn GmbH), 2 cm in diameter and made from conductive rubber, were placed via Ten 20 electrode paste (Weaver and Co., Aurora, CO, USA) on the scalp above the hand M1 in the left hemisphere and on the contralateral forehead (2 cm above the right orbit), respectively. The position of the anode was anatomically determined as the center of the ‘hand knob’^55^ projected on the scalp using the individual T1-weighted MRI and Brainsight neuronavigation system (Rogue Research Inc., Canada).

Stimulus duration was 10 min with fade-in and -out of 15 s in accordance with previous studies (e.g.,^17^^,^^56^). In the sham condition, electric current (1 mA) was applied only for the first 30 s with the same duration of feed-in and-out phases. During the simulation, participants were asked to be seated and to direct their attention around the right FDI muscle to enhance the effect of tDCS on corticospinal excitability.^45^ Each participant underwent all five conditions with a wash-out period of at least five days. The order of conditions was counterbalanced across participants according to a Latin square.

The experimental sessions took place either during morning (49 sessions, starting between 9:00a.m. and 11:30a.m., median: 10:30a.m.) or in the afternoon (56 sessions, starting between 12:30p.m. and 6:00p.m., median: 2:00p.m.). The sessions for single participants were not necessarily delivered at the same time of the day (1 participant was always stimulated in the morning, 2 always in the afternoon, and the remaining 18 took part in both morning and afternoon sessions).

#### TMS and MEP recordings

To evaluate corticospinal excitability, MEPs were elicited from the relaxed FDI muscle using Magstim stimulator (Magstim 200,^2^ Magstim Co. Ltd, the United Kingdom) with a figure-eight coil (70 mm in diameter). The magnetic coil was oriented at an angle of approximately 45° from the midline with its center on the scalp above the anatomically-determined center of the hand knob, such that the induced current was in the posterior–anterior (PA) or anterior–posterior (AP) directions. At the beginning of each experimental session, the resting motor threshold (RMT) was measured for both current directions. The RMT was defined as the lowest intensity that elicited MEPs with peak-to-peak amplitudes greater than 50 μV in at least 5 of 10 stimuli.^57^

A technical error was made during the experiments. Due to the error, the electromyogram amplifier model was set incorrectly to ‘model 1,2’ in the Brainsight software (Rogue Research Inc., Canada) instead of ‘model 3’, which was the model actually used. Due to the incorrect setting, the recorded MEP sizes were too small by a factor of 4444/13500, which might have resulted the measured RMT to be greater than the true RMT. This possible overestimation of the RMT concerned rare cases where the true MEP sizes were greater than 50 μV but less than 13500/4444×50 μV, which would be incorrectly classified as being below the RMT, depending on the experimenter’s judgment. In these cases, the TS intensity might have slightly exceeded the planned intensity of 120% of the true RMT. The effect of the deviation from the study protocol was expected to be small (see ‘limitations of the study’). To correct the error, the recorded MEP sizes were scaled with a factor of 13500/4444 for all subsequent analyses.

Twenty MEP signals were measured for each direction in four time windows: pre-tDCS baseline, and post-tDCS time windows starting at 0, 15 and 30 min after tDCS. The order of PA and AP stimulation was balanced across participants. Jitter was applied on the inter-stimulus interval to avoid anticipatory effects (mean ± SD of inter-stimulus interval: 4.7±0.8 s, minimum: 3.5 s). The electromyogram signals were amplified, bandpass filtered between 16 and 470 Hz, and sampled at 3 kHz. For each time window and direction, the MEP size was calculated as the mean peak-to-peak MEP amplitude over the twenty signals. The MEP latency was determined by calculating the mean signal over the twenty MEP traces and using cross-correlation in MATLAB (MathWorks Inc., Natick, MA) to find the delay of the mean MEP signal from that of the baseline of PA stimulation.

#### Generation of volume conductor models

The MR images were segmented to generate volume conductor models using an approach described previously.^58^ Briefly, white matter and gray matter surfaces were reconstructed using FreeSurfer software (version 7.1.1).^52^^,^^53^^,^^54^ Custom MATLAB scripts and manual processing were used for the reconstruction of the inner and outer surfaces of the skull and the outer surface of the head. It was ensured that the brain was separated from the skull by at least 0.5 mm.

We note that the CSF–skull and skull–scalp boundaries were affected by the chemical shift artifact in T1-weighted images, which made the skull thickness appear different in the T1 and T2 weighted images (see Figure S4). Therefore, the T2 weighted images were used for skull segmentation, as they produce a more realistic estimate of the skull thickness, as has been shown by a comparison with X-ray computed tomography images.^59^

After the generation of the surfaces, a model of the dura matter (1 mm thickness) was inserted between the CSF and skull compartments. The compartment between the dura mater and gray matter was segmented into CSF and blood by thresholding the T1 and T2 weighted image data and applying image processing methods, such as region growing and closure. Similarly, the skull was segmented into cortical bone and cancellous bone using the T2 weighted image data, and the compartment between the outer surface of the skull and the outer surface of the head was segmented into fat, muscle, and scalp.

To generate volume conductor models, the head was divided into a regular grid of 0.5 mm × 0.5 mm × 0.5 mm elements. The electric conductivity of each element was calculated from the proportions of tissues inside the element. For instance, if an element on the gray matter–CSF boundary consisted of 80% gray matter and 20% CSF, its conductivity was a weighted average of the gray matter and CSF conductivities. This approach for setting the element conductivities was used at the boundaries of white and gray matter, gray matter and CSF, CSF and dura, dura and compact bone, scalp and electrode paste, electrode paste and rubber, electrode paste and air, and electrode rubber and air. Any other boundaries were modeled using the conventional staircase approximation (see Figure S5B for an example).

As the method for generation of the volume conductor models can affect the estimated electric field values, alternative versions of all head models were generated from the T1-and T2-weighted images using charm,^59^ an automatic segmentation method. The default settings were used and no manual corrections were applied to the models generated using charm. To study the sensitivity of the findings to the segmentation method, all calculations were repeated for the alternative models. For these calculations, the models were upsampled to a grid of 0.5 mm × 0.5 mm × 0.5 mm elements.

For the volume conductor models, including the alternative models, the conductivity values assigned to the tissues and bodily fluids were identical to our previous study on tDCS dose response^24^ and were (unit: S/m): gray matter: 0.2, white matter: 0.14, CSF: 1.8, blood: 0.7, dura: 0.16, compact bone: 0.008, cancellous bone: 0.027, scalp: 0.08, fat: 0.08, muscle: 0.16, and eye: 1.5.

The electrical conductivity values are uncertain, and various estimates have been used in the literature.^60^ To assess the sensitivity of the findings to the conductivity values, the conductivities of the tissues with the highest (CSF) and lowest (bone) conductivities were varied. The conductivities of cortical and cancellous bone were modified by ±50%, approximately corresponding to the lowest and highest estimates in the literature.^60^ The CSF conductivity was varied by ±10%, giving values that are close to commonly used estimates of 1.654 S/m^61^ and 2 S/m.^62^

#### Modeling of stimulation electrodes

TDCS electrodes, including the rubber and a layer of paste, were inserted in the volume conductor models at the coordinates recorded using Brainsight. The dimensions of the electrode models were based on measurements using a digital caliper and are visualized in Figure S5A. A 2 mm thick layer of electrode paste was added between the scalp and the rubber. The radius of the paste layer was 1 mm larger than the radius of the rubber to simulate the paste application in the experiments. The electric current was fed to the electrodes through needle-shaped connectors located inside the electrode rubber, simulating the metal connectors of the real electrodes. For the finite-element simulations, block models of the needles were constructed by finding all elements that were penetrated by the needles. Dirichlet boundary conditions were applied at the nodes of the block models. The current density under the electrode is illustrated in Figure S5C.

The conductivities of electrode rubber and paste were determined based on measurements that were performed in rubber (N=9) and paste (N=6) samples at the frequency of 20 Hz using an impedance analyzer (E4990A, Keysight Technologies, Santa Rosa, CA). The obtained mean values were directly used in the volume conductor model. They and the standard deviations were 28.3±1.4 S/m for rubber and 0.504±0.005 S/m for the electrode paste.

#### Electric field modeling

The electric scalar potential φ generated by tDCS was modeled by solving the scalar potential equation ∇⋅σ∇φ=0 with Dirichlet boundary conditions at the anode and cathode and homogeneous Neumann boundary conditions elsewhere. The finite-element method with first-order 0.5 mm × 0.5 mm x 0.5 mm cubical elements and a geometric multigrid solver implemented in MATLAB^51^ were used for obtaining the numerical solution with a relative residual norm of $10^{-6}$. The electric field was obtained from $\vec{E}=-\nabla \varphi$ and scaled so that the current flowing into the head matched the desired stimulation current.

Our previous study indicated that the changes in the MEP size following tDCS are mediated by the normal component of the electric field at the activation site of TMS.^24^ Therefore, for statistical analysis, we calculated the normal component of the electric field at the average cortical activation site of the FDI muscle, $r_{\text{FDI}}=\left[-43,-11,60\right]$,^28^^,^^63^ which was mapped from the Montreal Neurological Institute (MNI) space to each participants’ brain (at the depth of 2 mm below the pial surface) using FreeSurfer. This electric field value is denoted by $E_{\text{FDI}}$.

To test the sensitivity of the results to the choice of the electric field outcome measure, we repeated the statistical analyses described below for additional outcome measures. The studied measures were: (1) ${\left|E\right|}_{\text{FDI}}$, the strength of the electric field at $r_{\text{FDI}}$; (2) $E_{\text{sphere}}$, the electric field strength averaged over the intersection of the gray matter of the precentral gyrus and a sphere with 3 cm diameter, centered at $r_{\text{FDI}}$; (3) $E_{\text{TMS}}$, the electric field strength averaged over the cortical region where the TMS-induced electric field strength was larger than 70% of the maximum; and (4) $E_{\text{FDI}}$ and ${\left|E\right|}_{\text{FDI}}$ when the location of $r_{\text{FDI}}$ was varied in each participant’s left hemisphere.

For (3), the electric fields induced by TMS were modeled using the finite element method with the same parameters as those used for modeling tDCS. The electric scalar potential was determined by solving $\nabla \cdot \sigma \nabla \varphi =-\nabla \cdot \sigma \frac{\partial \vec{A}}{\partial t}$, where $\vec{A}$ is the magnetic vector potential of the figure-of-eight coil.^28^ The boundary condition was $\vec{n}\cdot \left(\nabla \varphi +\frac{\partial \vec{A}}{\partial t}\right)=0$, where $\vec{n}$ is the normal vector on the body surface. The TMS electric field was determined from $\vec{E}=-\nabla \varphi -\frac{\partial \vec{A}}{\partial t}$. The electrical conductivity values used for modeling TMS differed slightly from those used for tDCS and were (unit: S/m)^28^: gray matter: 0.215, white matter: 0.142, CSF: 1.79, blood: 0.7, dura: 0.18, compact bone: 0.009, cancellous bone: 0.034, scalp: 0.43, fat: 0.15, muscle: 0.18, and eye: 1.5.

For (4), the electric field calculated in each participant’s left hemisphere (2 mm below the pial surface) was first mapped to the surface of the MNI template,^58^ and new $r_{\text{FDI}}$ locations were then selected on this template (see Figure S3).

### Quantification and statistical analysis

We used linear mixed effects models in MATLAB to investigate the effect of $E_{\text{FDI}}$, current, and other experimental factors on the normalized MEP size and MEP latency.

For all models, maximum likelihood was used to estimate the model coefficients. The generalized extreme Studentized deviate test for the model residuals was used to detect outliers, and if any were found, they were excluded from the model fit. Normal probability plots were investigated to verify the normality of residuals, and log transforms were used in cases where there were clear violations of normality. Likelihood ratio tests were used to investigate the statistical significance of the fixed effects by comparing the full model with an alternative model that excluded the fixed-effect predictor under investigation. The level of statistical significance was chosen as p=0.05. All reported p values are uncorrected for multiple comparisons, but we used the Benjamini-Hochberg procedure^64^ for controlling the false discovery rate (FDR) at a level of 0.05 whenever multiple tests were performed.

The details of the fitted models, including the formulas as well as all coefficient estimates, are provided in Tables S1–S5.

#### Effect of current on the normalized MEP size

To study the effect of stimulation current, we fitted a model where the dependent variable was the MEP size that was normalized to the baseline by dividing the MEP size with the value at the baseline. The normalized MEP size was further log transformed to ensure the normality of residuals.

The fixed effects were the stimulation current (sham, 0.5, 1, 1.5, or 2 mA as a categorical variable with five levels), TMS direction (PA or AP), time point (0, 15, or 30 min after tDCS), and all their interaction effects. Gender, its interaction with current, and session number (1–5) were also included as fixed effects.

As the time of day can affect the individual responses to tDCS,^44^ the time of day (morning or afternoon) and its interaction with stimulation current were included as fixed effects to control possible chronotype differences.

The normalized MEP size is affected by the baseline MEP size.^30^ To control for this effect and possible current-dependent differences, the logarithm of the baseline MEP size and its interaction with current were included as fixed effects.

The random effects were an intercept and slopes for stimulation current, TMS direction, time point, time of day, and baseline MEP for each participant. The interaction terms could not be included as random slopes due to the lack of data. Random correlations were dropped from the model, as the full model failed to converge.^65^ Furhermore, the random effect standard deviations for each stimulation current were assumed equal. The model is summarized in Table S1.

#### Effect of electric field on the normalized MEP size

Another model was used to study the relationship between the individual $E_{\text{FDI}}$ value and the logarithm of the normalized MEP. The model was otherwise similar to the model described above, but current was replaced by the first, second, and third order polynomials of $E_{\text{FDI}}$ (continuous variables). The rationale for using polynomial dependency was that the dependence of the MEP size on the electric field can be non-linear. The polynomials were orthonormal with respect to the observed values of $E_{\text{FDI}}$.^66^ The model is summarized in Table S2.

#### Effects of current or electric field on the absolute MEP size

Alternative linear mixed effects models were fitted to study the effects of current and electric field on the absolute (non-normalized) MEP sizes. The dependent variable was the MEP size, which was log-transformed as normal probability plots of the residuals indicated violations of normality.

The fixed effects were the time point (including the baseline time point), TMS direction, current or the electric field, and all their interaction terms. Session number, gender, and time of day were also included to control possible differences in the baseline MEP level. The random effects were by-subject intercepts and slopes for time point, direction, time of day, and session number. The models are summarised in Tables S3 and S4.

#### Effects of current on the MEP latency

The effects on the MEP latency were studied using a similar model to the effect-of-current model described above, but the dependent variable was the MEP latency difference from the baseline of PA stimulation, and thus, no terms featuring intercepts were included. The model is summarized in Table S5.

#### Estimation of variable importance

The variable importance of predictors was estimated with the permutation method^67^ using the coefficient of determination as the performance metric. Briefly, the values in the input dataset corresponding to a predictor of interest were permuted randomly, after which a new prediction was calculated using the permuted data, without refitting the model. The variable importance was calculated as the difference between the coefficients of determination calculated from the original and new predictions. This process was repeated 1000 times for each predictor term and the mean value was then calculated.

#### Visualization and confidence bounds

Partial dependence plots^68^ were used for visualization. Bias corrected and accelerated percentile method with 10000 bootstrap data samples was used to calculate 95% confidence bounds for the partial dependence plots and other quantities calculated from the plots.

## References

[1] Kuo M.-F., Paulus W., Nitsche M.A. (2014). Therapeutic effects of non-invasive brain stimulation with direct currents (tDCS) in neuropsychiatric diseases. Neuroimage.

[2] Priori A., Berardelli A., Rona S., Accornero N., Manfredi M. (1998). Polarization of the human motor cortex through the scalp. Neuroreport.

[3] Nitsche M.A., Paulus W. (2000). Excitability changes induced in the human motor cortex by weak transcranial direct current stimulation. J. Physiol. (Lond.).

[4] Jamil A., Nitsche M.A. (2017). What effect does tDCS have on the brain? Basic physiology of tDCS. Curr. Behav. Neurosci. Rep..

[5] Liebetanz D., Nitsche M.A., Tergau F., Paulus W. (2002). Pharmacological approach to the mechanisms of transcranial DC-stimulation-induced after-effects of human motor cortex excitability. Brain.

[6] Hummel F., Celnik P., Giraux P., Floel A., Wu W.-H., Gerloff C., Cohen L.G. (2005). Effects of non-invasive cortical stimulation on skilled motor function in chronic stroke. Brain.

[7] Hummel F., Cohen L.G. (2005). Improvement of motor function with noninvasive cortical stimulation in a patient with chronic stroke. Neurorehabilitation Neural Repair.

[8] Bastani A., Jaberzadeh S. (2013). Differential modulation of corticospinal excitability by different current densities of anodal transcranial direct current stimulation. PLoS One.

[9] Jamil A., Batsikadze G., Kuo H.-I., Labruna L., Hasan A., Paulus W., Nitsche M.A. (2017). Systematic evaluation of the impact of stimulation intensity on neuroplastic after-effects induced by transcranial direct current stimulation. J. Physiol. (Lond.).

[10] Ammann C., Lindquist M.A., Celnik P.A. (2017). Response variability of different anodal transcranial direct current stimulation intensities across multiple sessions. Brain Stimul..

[11] Batsikadze G., Moliadze V., Paulus W., Kuo M.-F., Nitsche M.A. (2013). Partially non-linear stimulation intensity-dependent effects of direct current stimulation on motor cortex excitability in humans. J. Physiol. (Lond.).

[12] Mosayebi Samani M., Agboada D., Jamil A., Kuo M.-F., Nitsche M.A. (2019). Titrating the neuroplastic effects of cathodal transcranial direct current stimulation (tDCS) over the primary motor cortex. Cortex.

[13] Kidgell D.J., Daly R.M., Young K., Lum J., Tooley G., Jaberzadeh S., Zoghi M., Pearce A.J. (2013). Different current intensities of anodal transcranial direct current stimulation do not differentially modulate motor cortex plasticity. Neural Plast..

[14] Ho K.-A., Taylor J.L., Chew T., Gálvez V., Alonzo A., Bai S., Dokos S., Loo C.K. (2016). The effect of transcranial direct current stimulation (tDCS) electrode size and current intensity on motor cortical excitability: Evidence from single and repeated sessions. Brain Stimul..

[15] Agboada D., Mosayebi Samani M., Jamil A., Kuo M.-F., Nitsche M.A. (2019). Expanding the parameter space of anodal transcranial direct current stimulation of the primary motor cortex. Sci. Rep..

[16] Tremblay S., Larochelle-Brunet F., Lafleur L.-P., El Mouderrib S., Lepage J.-F., Théoret H. (2016). Systematic assessment of duration and intensity of anodal transcranial direct current stimulation on primary motor cortex excitability. Eur. J. Neurosci..

[17] Chew T., Ho K.-A., Loo C.K. (2015). Inter- and intra-individual variability in response to transcranial direct current stimulation (tDCS) at varying current intensities. Brain Stimul..

[18] Esmaeilpour Z., Marangolo P., Hampstead B.M., Bestmann S., Galletta E., Knotkova H., Bikson M. (2018). Incomplete evidence that increasing current intensity of tDCS boosts outcomes. Brain Stimul..

[19] Laakso I., Tanaka S., Koyama S., De Santis V., Hirata A. (2015). Inter-subject variability in electric fields of motor cortical tDCS. Brain Stimul..

[20] Opitz A., Paulus W., Will S., Antunes A., Thielscher A. (2015). Determinants of the electric field during transcranial direct current stimulation. Neuroimage.

[21] Mosayebi-Samani M., Jamil A., Salvador R., Ruffini G., Haueisen J., Nitsche M.A. (2021). The impact of individual electrical fields and anatomical factors on the neurophysiological outcomes of tDCS: A TMS-MEP and MRI study. Brain Stimul..

[22] Antonenko D., Grittner U., Saturnino G., Nierhaus T., Thielscher A., Flöel A. (2021). Inter-individual and age-dependent variability in simulated electric fields induced by conventional transcranial electrical stimulation. Neuroimage.

[23] Evans C., Bachmann C., Lee J.S.A., Gregoriou E., Ward N., Bestmann S. (2020). Dose-controlled tDCS reduces electric field intensity variability at a cortical target site. Brain Stimul..

[24] Laakso I., Mikkonen M., Koyama S., Hirata A., Tanaka S. (2019). Can electric fields explain inter-individual variability in transcranial direct current stimulation of the motor cortex?. Sci. Rep..

[25] Ahn S., Fröhlich F. (2021). Pinging the brain with transcranial magnetic stimulation reveals cortical reactivity in time and space. Brain Stimul..

[26] Mikkonen M., Laakso I., Tanaka S., Hirata A. (2020). Cost of focality in TDCS: Interindividual variability in electric fields. Brain Stimul..

[27] Rawji V., Ciocca M., Zacharia A., Soares D., Truong D., Bikson M., Rothwell J., Bestmann S. (2018). tDCS changes in motor excitability are specific to orientation of current flow. Brain Stimul..

[28] Laakso I., Murakami T., Hirata A., Ugawa Y. (2018). Where and what TMS activates: experiments and modeling. Brain Stimul..

[29] Corp D.T., Bereznicki H.G.K., Clark G.M., Youssef G.J., Fried P.J., Jannati A., Davies C.B., Gomes-Osman J., Stamm J., Chung S.W. (2020). Large-scale analysis of interindividual variability in theta-burst stimulation data: Results from the ‘big TMS data collaboration. Brain Stimul..

[30] Corp D.T., Bereznicki H.G.K., Clark G.M., Youssef G.J., Fried P.J., Jannati A., Davies C.B., Gomes-Osman J., Kirkovski M., Albein-Urios N. (2021). Large-scale analysis of interindividual variability in single and paired-pulse TMS data. Clin. Neurophysiol..

[31] Vasu S.O., Kaphzan H. (2021). The role of sodium channels in direct current stimulation—axonal perspective. Cell Rep..

[32] Bikson M., Rahman A., Datta A., Fregni F., Merabet L. (2012). High-resolution modeling assisted design of customized and individualized transcranial direct current stimulation protocols. Neuromodulation.

[33] Caulfield K.A., Badran B.W., DeVries W.H., Summers P.M., Kofmehl E., Li X., Borckardt J.J., Bikson M., George M.S. (2020). Transcranial electrical stimulation motor threshold can estimate individualized tDCS dosage from reverse-calculation electric-field modeling. Brain Stimul..

[34] Van Hoornweder S., Nuyts M., Frieske J., Verstraelen S., Meesen R.L.J., Caulfield K.A. (2023). Outcome measures for electric field modeling in tES and TMS: A systematic review and large-scale modeling study. Neuroimage.

[35] Shinde A.B., Lerud K.D., Munsch F., Alsop D.C., Schlaug G. (2021). Effects of tDCS dose and electrode montage on regional cerebral blood flow and motor behavior. Neuroimage.

[36] Hsu G., Shereen A.D., Cohen L.G., Parra L.C. (2023). Robust enhancement of motor sequence learning with 4 mA transcranial electric stimulation. Brain Stimul..

[37] Di Lazzaro V., Oliviero A., Saturno E., Pilato F., Insola A., Mazzone P., Profice P., Tonali P., Rothwell J.C. (2001). The effect on corticospinal volleys of reversing the direction of current induced in the motor cortex by transcranial magnetic stimulation. Exp. Brain Res..

[38] Di Lazzaro V., Profice P., Ranieri F., Capone F., Dileone M., Oliviero A., Pilato F. (2012). I-wave origin and modulation. Brain Stimul..

[39] Siebner H.R., Funke K., Aberra A.S., Antal A., Bestmann S., Chen R., Classen J., Davare M., Di Lazzaro V., Fox P.T. (2022). Transcranial magnetic stimulation of the brain: What is stimulated? – a consensus and critical position paper. Clin. Neurophysiol..

[40] Evans C., Zich C., Lee J.S.A., Ward N., Bestmann S. (2022). Inter-individual variability in current direction for common tDCS montages. Neuroimage.

[41] Kiernan M.C., Cikurel K., Bostock H. (2001). Effects of temperature on the excitability properties of human motor axons. Brain.

[42] Tremblay F., Remaud A., Mekonnen A., Gholami-Boroujeny S., Racine K.É., Bolic M. (2015). Lasting depression in corticomotor excitability associated with local scalp cooling. Neurosci. Lett..

[43] Wright K.P., Hull J.T., Czeisler C.A. (2002). Relationship between alertness, performance, and body temperature in humans. Am. J. Physiol..

[44] Salehinejad M.A., Wischnewski M., Ghanavati E., Mosayebi-Samani M., Kuo M.-F., Nitsche M.A. (2021). Cognitive functions and underlying parameters of human brain physiology are associated with chronotype. Nat. Commun..

[45] Yamaguchi T., Moriya K., Tanabe S., Kondo K., Otaka Y., Tanaka S. (2020). Transcranial direct-current stimulation combined with attention increases cortical excitability and improves motor learning in healthy volunteers. J. NeuroEng. Rehabil..

[46] López-Alonso V., Fernández-Del-Olmo M., Costantini A., Gonzalez-Henriquez J.J., Cheeran B. (2015). Intra-individual variability in the response to anodal transcranial direct current stimulation. Clin. Neurophysiol..

[47] Turi Z., Ambrus G.G., Ho K.-A., Sengupta T., Paulus W., Antal A. (2014). When size matters: Large electrodes induce greater stimulation-related cutaneous discomfort than smaller electrodes at equivalent current density. Brain Stimul..

[48] Kuo H.-I., Bikson M., Datta A., Minhas P., Paulus W., Kuo M.-F., Nitsche M.A. (2013). Comparing cortical plasticity induced by conventional and high-definition 4 ×1 ring tDCS: a neurophysiological study. Brain Stimul..

[49] Sasia B., Cacciamani L. (2021). High-definition transcranial direct current stimulation of the lateral occipital cortex influences figure-ground perception. Neuropsychologia.

[50] Gbadeyan O., Steinhauser M., McMahon K., Meinzer M. (2016). Safety, tolerability, blinding efficacy and behavioural effects of a novel MRI-compatible, high-definition tDCS set-up. Brain Stimul..

[51] Laakso I., Hirata A. (2012). Fast multigrid-based computation of the induced electric field for transcranial magnetic stimulation. Phys. Med. Biol..

[52] Dale A.M., Fischl B., Sereno M.I. (1999). Cortical surface-based analysis. i. segmentation and surface reconstruction. Neuroimage.

[53] Fischl B., Sereno M.I., Dale A.M. (1999). Cortical surface-based analysis. ii: Inflation, flattening, and a surface-based coordinate system. Neuroimage.

[54] Fischl B., Sereno M.I., Tootell R.B., Dale A.M. (1999). High-resolution intersubject averaging and a coordinate system for the cortical surface. Hum. Brain Mapp..

[55] Yousry T.A., Schmid U.D., Alkadhi H., Schmidt D., Peraud A., Buettner A., Winkler P. (1997). Localization of the motor hand area to a knob on the precentral gyrus. a new landmark. Brain.

[56] Wiethoff S., Hamada M., Rothwell J.C. (2014). Variability in response to transcranial direct current stimulation of the motor cortex. Brain Stimul..

[57] Rossini P.M., Burke D., Chen R., Cohen L.G., Daskalakis Z., Di Iorio R., Di Lazzaro V., Ferreri F., Fitzgerald P.B., George M.S. (2015). Non-invasive electrical and magnetic stimulation of the brain, spinal cord, roots and peripheral nerves: Basic principles and procedures for routine clinical and research application. an updated report from an I.F.C.N. committee. Clin. Neurophysiol..

[58] Laakso I., Tanaka S., Mikkonen M., Koyama S., Sadato N., Hirata A. (2016). Electric fields of motor and frontal tDCS in a standard brain space: A computer simulation study. Neuroimage.

[59] Puonti O., Van Leemput K., Saturnino G.B., Siebner H.R., Madsen K.H., Thielscher A. (2020). Accurate and robust whole-head segmentation from magnetic resonance images for individualized head modeling. Neuroimage.

[60] Saturnino G.B., Madsen K.H., Thielscher A. (2019). Electric field simulations for transcranial brain stimulation using FEM: an efficient implementation and error analysis. J. Neural. Eng..

[61] Wagner T.A., Zahn M., Grodzinsky A.J., Pascual-Leone A. (2004). Three-dimensional head model simulation of transcranial magnetic stimulation. IEEE Trans. Biomed. Eng..

[62] Parazzini M., Fiocchi S., Rossi E., Paglialonga A., Ravazzani P. (2011). Transcranial direct current stimulation: Estimation of the electric field and of the current density in an anatomical human head model. IEEE Trans. Biomed. Eng..

[63] Volz L.J., Hamada M., Rothwell J.C., Grefkes C. (2015). What makes the muscle twitch: Motor system connectivity and TMS-induced activity. Cerebr. Cortex.

[64] Benjamini Y., Hochberg Y. (1995). Controlling the false discovery rate: a practical and powerful approach to multiple testing. J. R. Stat. Soc. Series B.

[65] Barr D.J., Levy R., Scheepers C., Tily H.J. (2013). Random effects structure for confirmatory hypothesis testing: Keep it maximal. J. Mem. Lang..

[66] Smyth G.K., Armitage P., Colton T. (1998). Encyclopedia of Biostatistics.

[67] Greenwell B., Boehmke B. (2020). Variable importance plots—an introduction to the vip package. The R Journal.

[68] Friedman J.H. (2001). Greedy function approximation: a gradient boosting machine. Ann. Stat..

