# Supplementary material for: Small effects of electric field on motor cortical excitability following anodal tDCS
Source: iScience. 2024 Feb 1;27(2):108967. doi: 10.1016/j.isci.2024.108967 (PMC10863330; doi:10.1016/j.isci.2024.108967)
Supplement: Document S1. Figures S1–S5 and Tables S1–S5 [file mmc1.pdf]

iScience, Volume *27*

Supplemental information

Small effects of electric field on motor cortical excitability following anodal tDCS

Ilkka Laakso, Keisuke Tani, Jose Gomez-Tames, Akimasa Hirata, and Satoshi Tanaka

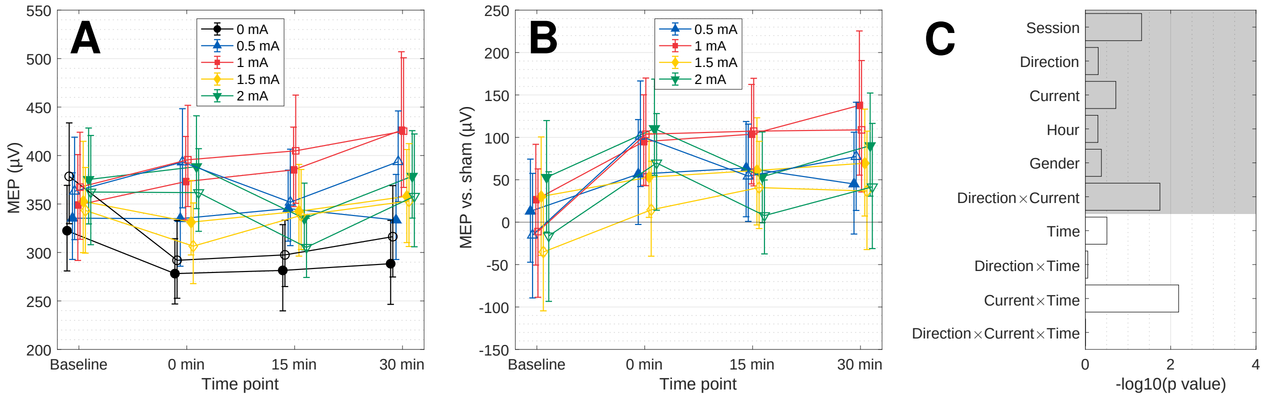

Figure S1: Visualization of the effects on the absolute MEP size, related to Figure 1. **A.** Partial dependence of the MEP size on the stimulation current, time point, and TMS direction. Filled and empty markers indicate PA- and AP-TMS, respectively. Error bars are 95% confidence intervals obtained using bootstrapping. **B.** Partial dependence of the difference in the MEP size from that of the sham condition. **C.** P-values of likelihood ratio tests. Baseline terms that are unrelated to the effect of the intervention are highlighted in grey. The effect of time on the MEP size depended on the stimulation current ($\chi^2(12) = 27.5$, $p = 0.007$) but was not statistically significant with a false discovery rate of 0.05 due to the inclusion of the baseline terms in the Benjamini-Hochberg procedure.

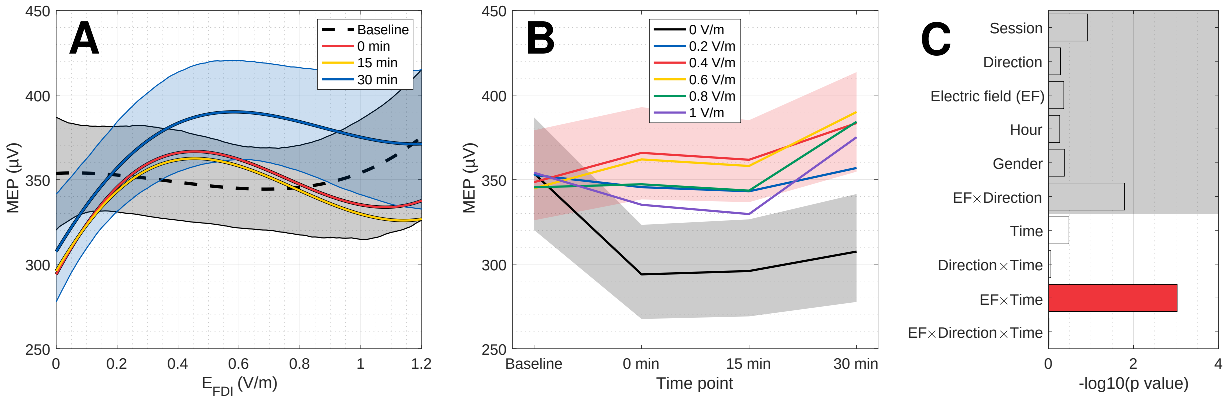

Figure S2: Effects of electric field and time point on the absolute MEP sizes, related to Figure 4. **A.** Partial dependence of the MEP size as a function of the electric field for each time point, showing a lack of effect of the electric field at the baseline compared with post-tDCS time points. **B.** Partial dependence of the MEP size as a function of time point for various values of the electric field, showing a difference in the time-dependency of the MEP size between active conditions and sham (0 V/m). In both plots, the shaded areas indicate 95% confidence bounds. For clarity, the confidence bounds are shown only for two quantities in each plot. **C.** P-values of likelihood ratio tests. Baseline terms unrelated to the effect of the intervention are highlighted in grey. Coloured bars indicate statistically significant effects with false discovery rate of 0.05. The effect of time on the absolute MEP size depended on the E_{FDI} value ($\chi^2(9) = 28$, $p = 0.0009$).

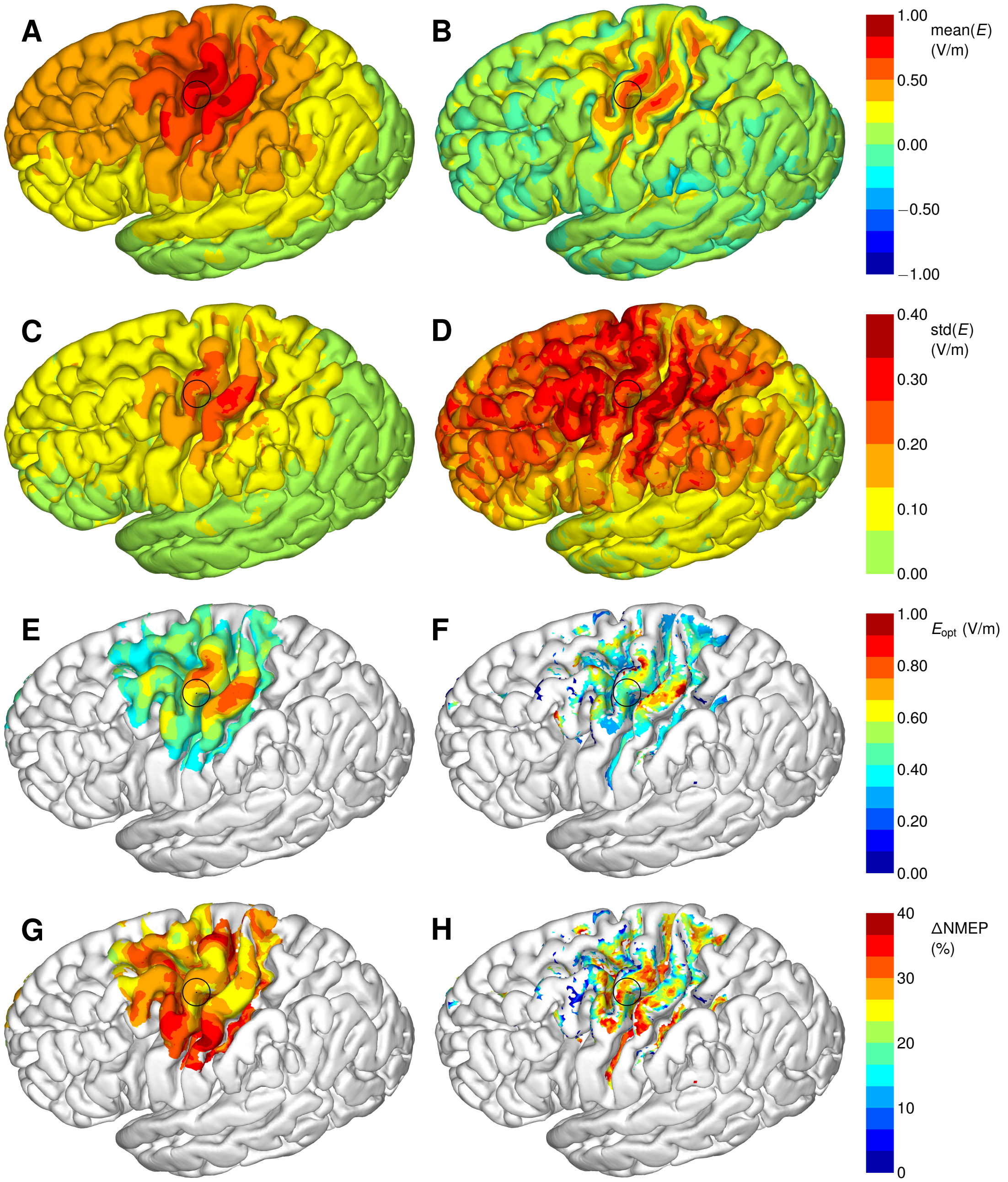

Figure S3: Sensitivity analysis for the location of interest, related to Table 1. **A, B.** Mean electric field strength (A) and inner normal component (B) registered to the template brain. The mean is calculated over all 21 participants. **C, D.** Standard deviation of the electric field strength and normal component. **E, F.** The optimal electric field value (E_{opt}) estimated using a linear mixed effects model fitted using the electric field data of each location on the template brain. Locations with weak electric fields (mean value smaller than its 90th percentile over the whole hemisphere), no E_{opt} value, or locations where the model fit failed to converge are shown in light grey. **G, H.** Predicted increase in the normalized MEP size versus sham stimulation for the respective E_{opt} values. In each panel, circles mark the location of interest, $r_{FDI} = [-41, -7, 63]$ in standard coordinates, used in the main text.

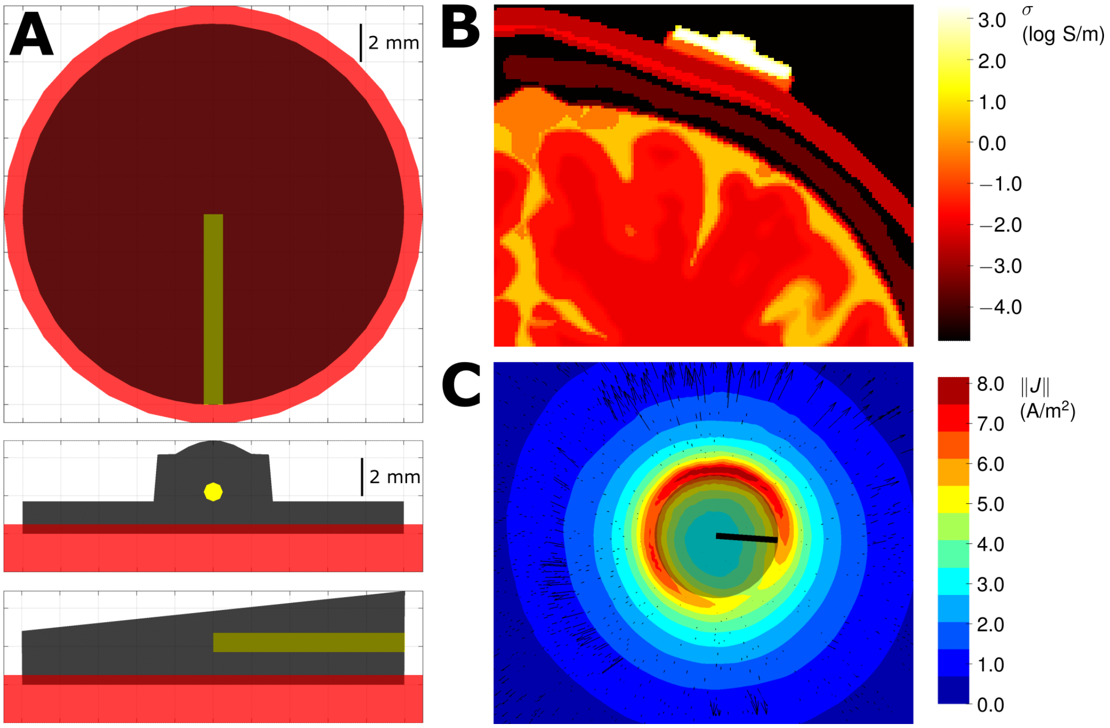

Figure S4: Electrode model, related to STAR Methods. **A.** Electrode dimensions. Transparent red: Electrode paste, transparent black: rubber, and yellow: metal connector. **B.** An electrode model placed in the finite element volume conductor model (coronal slice). Each pixel corresponds to one element and the colour scale indicates the conductivity value (log transformed) of the elements. **C.** Calculated current density at a depth of 1 mm below the scalp surface in a representative subject for a stimulation current of 1 mA. Shading and the black line segment show the electrode rubber and the metal connector, respectively.

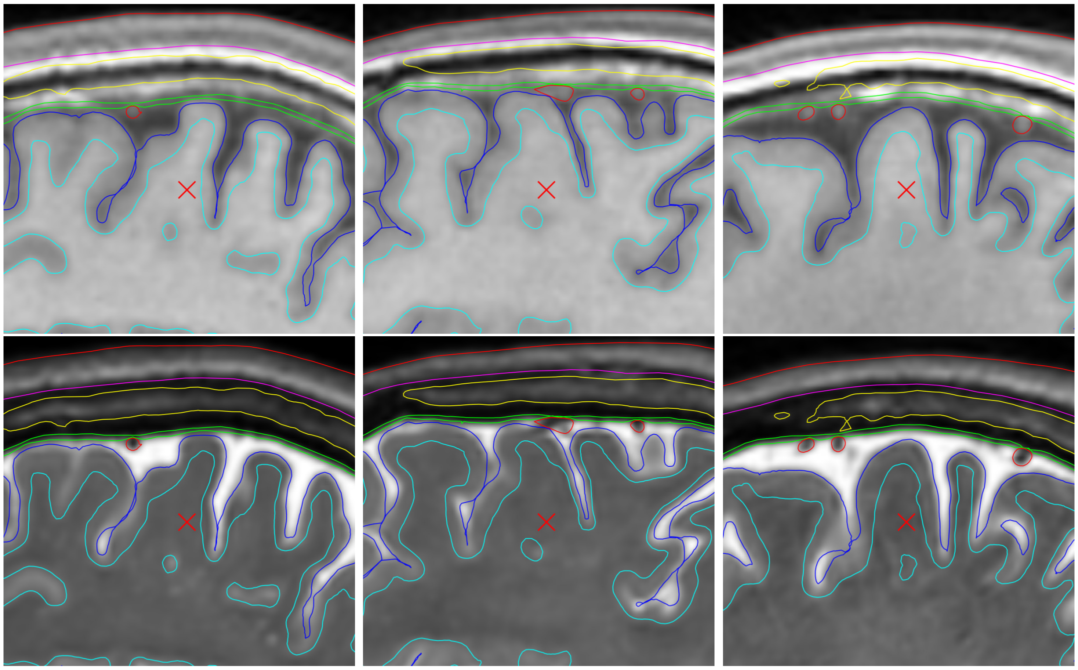

Figure S5: Examples of segmented MR images, related to STAR Methods. Sagittal cross-sections are shown for the surfaces of the white matter (cyan), grey matter (blue), the largest blood vessels (red), dura mater (green), cancellous bone (yellow), skull (magenta), and scalp (red) overlaid on T1 (top row) and T2 (bottom row) weighted MR images. Columns show three exemplary participants. The red cross indicates the anatomical hand knob target used for positioning the electrodes and the TMS coil using a neuronavigation system.

Table S1: Linear mixed effects model of the effects of current on the normalized MEP size, related to STAR Methods.

```
Linear mixed-effects model fit by ML

Model information:
    Number of observations            629
    Fixed effects coefficients         49
    Random effects coefficients       210
    Covariance parameters               8

Formula:
    logNMEP ~ logBaseMEP*Current + Session + Gender*Current + Hour*Current + Direction*Time*Current ...
        + (1 + logBaseMEP + Direction + Hour + Time|Subject) + (Current|Subject)

Model fit statistics:
    AIC       BIC       LogLikelihood    Deviance
    455.01    708.32    -170.5           341.01

Fixed effects coefficients (95% CIs):
    Name                                          Estimate      SE          tStat       DF     pValue        Lower        Upper
    {'(Intercept)'                       }         -0.039358    0.071574    -0.54989    580        0.5826     -0.17993      0.10122
    {'Session_1'                         }         -0.037935    0.048294    -0.78549    580       0.43249     -0.13279     0.056918
    {'Session_2'                         }          0.070038    0.051879        1.35    580       0.17753    -0.031856      0.17193
    {'Session_3'                         }         -0.089752    0.049461     -1.8146    580        0.0701      -0.1869     0.007392
    {'Session_4'                         }          0.022855    0.049616     0.46064    580       0.64523    -0.074594       0.1203
    {'Direction_PA'                      }         0.0058813    0.015208     0.38673    580        0.6991    -0.023987      0.03575
    {'Time_T00'                          }         -0.010564    0.018762    -0.56309    580       0.57359    -0.047413     0.026284
    {'Time_T15'                          }         -0.028852    0.017148     -1.6826    580      0.092991    -0.062531    0.0048264
    {'Current_0'                         }           -0.1739    0.045414     -3.8292    580    0.00014254      -0.2631    -0.084705
    {'Current_0.5'                       }          0.025638    0.045797     0.55982    580       0.57582    -0.064311      0.11559
    {'Current_1'                         }           0.12531    0.045277      2.7676    580     0.0058275     0.036381      0.21423
    {'Current_1.5'                       }         0.0029929    0.043926    0.068136    580        0.9457    -0.083281     0.089267
    {'logBaseMEP'                        }          -0.55928    0.057412     -9.7415    580    7.1913e-21     -0.67205     -0.44652
    {'Hour_Morning'                      }        -0.0088609    0.033354    -0.26566    580       0.79059     -0.07437     0.056649
    {'Gender_M'                          }          0.060081    0.071335     0.84224    580           0.4    -0.080025      0.20019
    {'Direction_PA:Time_T00'             }        -0.0010083    0.014534    -0.06938    580       0.94471    -0.029553     0.027536
    {'Direction_PA:Time_T15'             }         0.0087595    0.014555      0.6018    580       0.54754    -0.019828     0.037347
    {'Direction_PA:Current_0'            }         0.0032415    0.021631     0.14985    580       0.88093    -0.039244     0.045727
    {'Direction_PA:Current_0.5'          }         -0.036535    0.021119       -1.73    580      0.084166    -0.078014    0.0049435
    {'Direction_PA:Current_1'            }         -0.010799     0.02121    -0.50912    580       0.61086    -0.052457      0.03086
    {'Direction_PA:Current_1.5'          }          0.014359    0.021094      0.6807    580       0.49633    -0.027071     0.055788
    {'Time_T00:Current_0'                }         -0.013951    0.029056    -0.48013    580       0.63132    -0.071018     0.043117
    {'Time_T15:Current_0'                }          0.019766    0.029067     0.68003    580       0.49676    -0.037323     0.076856
    {'Time_T00:Current_0.5'              }          0.024579    0.029056     0.84593    580       0.39794    -0.032488     0.081647
    {'Time_T15:Current_0.5'              }         0.0025196    0.029067    0.086682    580       0.93095     -0.05457     0.059609
    {'Time_T00:Current_1'                }         -0.032721    0.029056     -1.1261    580       0.26057    -0.089789     0.024347
    {'Time_T15:Current_1'                }          0.013093    0.029067     0.45043    580       0.65257    -0.043997     0.070182
    {'Time_T00:Current_1.5'              }         -0.047496    0.029111     -1.6316    580       0.10331     -0.10467    0.0096792
    {'Time_T15:Current_1.5'              }           0.03561    0.029286       1.216    580        0.2245    -0.021909     0.093129
    {'Current_0:logBaseMEP'              }          0.054302    0.074195     0.73188    580       0.46454    -0.091422      0.20003
    {'Current_0.5:logBaseMEP'            }          0.052226    0.077995     0.66961    580       0.50337     -0.10096      0.20541
    {'Current_1:logBaseMEP'              }          0.090482    0.089513      1.0108    580       0.31252    -0.085327      0.26629
    {'Current_1.5:logBaseMEP'            }          -0.07664    0.070271     -1.0906    580       0.27589     -0.21466     0.061377
    {'Current_0:Hour_Morning'            }         -0.078144    0.053502     -1.4606    580       0.14467     -0.18323     0.026937
    {'Current_0.5:Hour_Morning'          }          0.041685     0.04899     0.85088    580       0.39519    -0.054535       0.1379
    {'Current_1:Hour_Morning'            }         -0.061896    0.053272     -1.1619    580       0.24576     -0.16653     0.042733
    {'Current_1.5:Hour_Morning'          }          0.015876    0.051395      0.3089    580       0.75751    -0.085067      0.11682
    {'Current_0:Gender_M'                }          -0.10244    0.048547     -2.1101    580      0.035279     -0.19779   -0.0070887
    {'Current_0.5:Gender_M'              }          0.043035    0.045486      0.9461    580       0.34449    -0.046303      0.13237
    {'Current_1:Gender_M'                }         -0.013873    0.044583    -0.31118    580       0.75578     -0.10144     0.073691
    {'Current_1.5:Gender_M'              }          0.033916    0.046941     0.72253    580       0.47026    -0.058279      0.12611
    {'Direction_PA:Time_T00:Current_0'   }         0.0095212    0.029056     0.32768    580       0.74327    -0.047547     0.066589
    {'Direction_PA:Time_T15:Current_0'   }         -0.003781    0.029067    -0.13008    580       0.89655     -0.06087     0.053308
    {'Direction_PA:Time_T00:Current_0.5'}          -0.021831    0.029056    -0.75133    580       0.45276    -0.078898     0.035237
    {'Direction_PA:Time_T15:Current_0.5'}           0.039451    0.029067      1.3572    580       0.17524    -0.017639      0.09654
    {'Direction_PA:Time_T00:Current_1'   }         -0.010605    0.029056    -0.36499    580       0.71525    -0.067673     0.046463
    {'Direction_PA:Time_T15:Current_1'   }         -0.015988    0.029067    -0.55005    580        0.5825    -0.073078     0.041101
    {'Direction_PA:Time_T00:Current_1.5'}           0.022945    0.029111     0.78821    580        0.4309     -0.03423     0.080121
    {'Direction_PA:Time_T15:Current_1.5'}          -0.020665    0.029286    -0.70565    580       0.48069    -0.078185     0.036854

Random effects covariance parameters (95% CIs):
Group: Subject (21 Levels)
    Name1                   Name2                   Type           Estimate    Lower       Upper
    {'(Intercept)' }        {'(Intercept)' }        {'std'}         0.31434     0.22353     0.44205
    {'Direction_PA'}        {'Direction_PA'}        {'std'}        0.049397    0.024744    0.098613
    {'Time_T00'    }        {'Time_T00'    }        {'std'}        0.054371     0.02752     0.10742
    {'Time_T15'    }        {'Time_T15'    }        {'std'}        0.041542    0.015585     0.11073
    {'logBaseMEP'  }        {'logBaseMEP'  }        {'std'}         0.14013    0.048993     0.40079
    {'Hour_Morning'}        {'Hour_Morning'}        {'std'}        0.069599    0.019875     0.24373

Group: Subject (21 Levels)
    Name1                Name2                Type           Estimate    Lower      Upper
    {'Current_0'}        {'Current_0'}        {'std'}        0.16012     0.12252    0.20926

Group: Error
    Name               Estimate    Lower      Upper
    {'Res Std'}        0.25781     0.24134    0.27541
```

Notes:

- Categorical predictors were modelled using effects coding.
- Diagonal covariance matrix was used for the first random effect vector and an isotropic covariance matrix for the second random effect vector.
- The logarithm of the baseline MEP was centred by subtracting its mean value (5.8762).

Table S2: Linear mixed effects model of the effects of electric field on the normalized MEP size, related to STAR Methods.

Linear mixed-effects model fit by ML

Model information:

Number of observations	629
Fixed effects coefficients	40
Random effects coefficients	189
Covariance parameters	8

Formula:

```
logNMEP ~ Session + Gender*p1E + Gender*p2E + Gender*p3E + logBaseMEP*p1E + logBaseMEP*p2E + logBaseMEP*p3E ...
    + Hour*p1E + Hour*p2E + Hour*p3E + Time*Direction*p1E + Time*Direction*p2E + Time*Direction*p3E ...
    + (1 + logBaseMEP + Hour + Time + Direction|Subject) + (p1E + p2E + p3E|Subject)
```

Model fit statistics:

AIC	BIC	LogLikelihood	Deviance
455.14	668.46	-179.57	359.14

Fixed effects coefficients (95% CIs):

Name	Estimate	SE	tStat	DF	pValue	Lower	Upper
{'(Intercept)' }	-0.033574	0.070514	-0.47613	589	0.63416	-0.17206	0.10492
{'Session_1' }	-0.10467	0.03635	-2.8796	589	0.0041267	-0.17606	-0.033281
{'Session_2' }	-0.00085285	0.035106	-0.024294	589	0.98063	-0.0698	0.068095
{'Session_3' }	-0.0028089	0.034527	-0.081356	589	0.93519	-0.070619	0.065001
{'Session_4' }	0.041579	0.036436	1.1411	589	0.25428	-0.029982	0.11314
{'Direction_PA' }	0.016408	0.01239	1.3243	589	0.18592	-0.0079259	0.040741
{'Time_T00' }	-0.010646	0.019112	-0.55702	589	0.57772	-0.048181	0.02689
{'Time_T15' }	-0.0286	0.017889	-1.5988	589	0.1104	-0.063734	0.0065327
{'logBaseMEP' }	-0.47494	0.076522	-6.2065	589	1.0221e-09	-0.62523	-0.32465
{'Hour_Morning' }	-0.0038159	0.038888	-0.098124	589	0.92187	-0.080193	0.072561
{'Gender_M' }	0.056471	0.070172	0.80476	589	0.42128	-0.081346	0.19429
{'p1E' }	2.4865	1.0985	2.2635	589	0.02397	0.32898	4.644
{'p2E' }	2.0269	1.2633	1.6045	589	0.10914	-0.45414	4.5079
{'p3E' }	3.9591	1.0238	3.8669	589	0.00012251	1.9483	5.9699
{'Direction_PA:Time_T00' }	-0.0011094	0.015218	-0.0729	589	0.94191	-0.030997	0.028778
{'Direction_PA:Time_T15' }	0.0089991	0.015238	0.59056	589	0.55504	-0.020929	0.038927
{'Direction_PA:p1E' }	0.16972	0.30305	0.56005	589	0.57566	-0.42547	0.76492
{'Time_T00:p1E' }	0.45272	0.39429	1.1482	589	0.25137	-0.32168	1.2271
{'Time_T15:p1E' }	-0.37609	0.39146	-0.96075	589	0.33707	-1.1449	0.39273
{'logBaseMEP:p1E' }	-2.785	1.2939	-2.1525	589	0.031764	-5.3261	-0.24389
{'Hour_Morning:p1E' }	0.36037	1.0069	0.35788	589	0.72056	-1.6173	2.338
{'Gender_M:p1E' }	0.87997	1.0558	0.83351	589	0.4049	-1.1935	2.9535
{'Direction_PA:p2E' }	-0.16115	0.30735	-0.52431	589	0.60026	-0.76479	0.44249
{'Time_T00:p2E' }	0.80819	0.39227	2.0603	589	0.039811	0.037766	1.5786
{'Time_T15:p2E' }	0.059154	0.38993	0.15171	589	0.87947	-0.70666	0.82497
{'logBaseMEP:p2E' }	-1.1061	1.5938	-0.69404	589	0.48793	-4.2363	2.024
{'Hour_Morning:p2E' }	1.5865	1.1056	1.435	589	0.15183	-0.58489	3.7579
{'Gender_M:p2E' }	-1.1462	1.1701	-0.97956	589	0.32771	-3.4444	1.1519
{'Direction_PA:p3E' }	-0.44048	0.30962	-1.4227	589	0.15536	-1.0486	0.1676
{'Time_T00:p3E' }	0.5616	0.38971	1.4411	589	0.15009	-0.20378	1.327
{'Time_T15:p3E' }	0.035502	0.3879	0.091522	589	0.92711	-0.72634	0.79734
{'logBaseMEP:p3E' }	-0.14155	1.5903	-0.089011	589	0.9291	-3.2648	2.9817
{'Hour_Morning:p3E' }	1.589	0.95133	1.6702	589	0.095403	-0.27946	3.4574
{'Gender_M:p3E' }	-1.1902	1.0315	-1.1539	589	0.24902	-3.2161	0.83564
{'Direction_PA:Time_T00:p1E'}	-0.066807	0.38171	-0.17502	589	0.86112	-0.81649	0.68287
{'Direction_PA:Time_T15:p1E'}	-0.33135	0.38202	-0.86735	589	0.38611	-1.0816	0.41895
{'Direction_PA:Time_T00:p2E'}	0.04753	0.38171	0.12452	589	0.90095	-0.70215	0.79721
{'Direction_PA:Time_T15:p2E'}	-0.19371	0.38202	-0.50707	589	0.6123	-0.94399	0.55658
{'Direction_PA:Time_T00:p3E'}	0.27994	0.38176	0.73329	589	0.46367	-0.46984	1.0297
{'Direction_PA:Time_T15:p3E'}	0.11266	0.38187	0.29501	589	0.76809	-0.63734	0.86265

Random effects covariance parameters (95% CIs):

Group: Subject (21 Levels)

Name1	Name2	Type	Estimate	Lower	Upper
{'(Intercept)' }	{'(Intercept)' }	{'std'}	0.29657	0.20634	0.42625
{'Direction_PA'}	{'Direction_PA'}	{'std'}	0.022127	0.0014318	0.34197
{'Time_T00' }	{'Time_T00' }	{'std'}	0.052983	0.025053	0.11205
{'Time_T15' }	{'Time_T15' }	{'std'}	0.042938	0.015289	0.12059
{'logBaseMEP' }	{'logBaseMEP' }	{'std'}	0.28429	0.184	0.43925
{'Hour_Morning'}	{'Hour_Morning'}	{'std'}	0.13712	0.0822	0.22872

Group: Subject (21 Levels)

Name1	Name2	Type	Estimate	Lower	Upper
{'p1E'}	{'p1E'}	{'std'}	2.4966	1.7899	3.4823

Group: Error

Name	Estimate	Lower	Upper
{'Res Std'}	0.26997	0.25295	0.28812

Notes:

- Categorical predictors were modelled using effects coding.
- Diagonal covariance matrix was used for the first random effect vector and an isotropic covariance matrix for the second random effect vector.
- The orthonormal polynomials with respect to the observed values of E_{FDI} were:

$$p_1(x) = 0.0860x - 0.0483 \quad (1)$$

$$p_2(x) = 0.1406x^2 - 0.2102x + 0.0435 \quad (2)$$

$$p_3(x) = 0.2250x^3 - 0.5991x^2 + 0.3780x - 0.0340. \quad (3)$$

- The logarithm of the baseline MEP was centred by subtracting its mean value (5.8762).

Table S3: Linear mixed effects model of the effects of current on the absolute MEP size, related to STAR Methods.

```
Linear mixed-effects model fit by ML

Model information:
    Number of observations            839
    Fixed effects coefficients         46
    Random effects coefficients       210
    Covariance parameters               8

Formula:
    logMEP ~ Session + Gender + Hour + Direction*Time*Current + (1 + Direction + Time + Hour|Subject) + (Session|Subject)

Model fit statistics:
    AIC       BIC       LogLikelihood    Deviance
    709.23    964.77    -300.62          601.23

Fixed effects coefficients (95% CIs):
    Name                                          Estimate      SE          tStat       DF     pValue        Lower          Upper
    {'(Intercept)'                       }             5.847    0.090332      64.728    793    1.0169e-318        5.6697        6.0243
    {'Session_1'                         }           0.10488    0.053493      1.9606    793        0.05027    -0.00012382       0.20989
    {'Session_2'                         }         0.0077943    0.054234     0.14372    793        0.88576     -0.098665        0.11425
    {'Session_3'                         }          -0.14412    0.053847     -2.6765    793      0.0075927      -0.24982     -0.038423
    {'Session_4'                         }         0.0019703    0.052697     0.03739    793        0.97018      -0.10147       0.10541
    {'Direction_PA'                      }         -0.013808    0.020277    -0.68094    793        0.49611     -0.053611      0.025996
    {'Time_Tbase'                        }          0.016859     0.03142     0.53657    793        0.59172     -0.044818      0.078536
    {'Time_T00'                          }         -0.016267    0.023982    -0.67828    793        0.49779     -0.063343       0.03081
    {'Time_T15'                          }         -0.034307      0.0206     -1.6654    793       0.096234     -0.074744     0.0061304
    {'Current_0'                         }          -0.11341    0.056205     -2.0177    793       0.043956      -0.22373    -0.0030784
    {'Current_0.5'                       }          0.010309    0.055019     0.18737    793        0.85142     -0.097692       0.11831
    {'Current_1'                         }           0.10634    0.054342      1.9569    793       0.050712   -0.00033041       0.21301
    {'Current_1.5'                       }         -0.020553    0.053211    -0.38626    793        0.69941        -0.125      0.083897
    {'Hour_Morning'                      }         -0.024412    0.036394    -0.67078    793        0.50256     -0.095851      0.047027
    {'Gender_M'                          }           0.07339    0.090312     0.81262    793        0.41668      -0.10389       0.25067
    {'Direction_PA:Time_Tbase'           }        -0.0091461    0.016585    -0.55145    793        0.58148     -0.041703       0.02341
    {'Direction_PA:Time_T00'             }         0.0019577    0.016585     0.11804    793        0.90607     -0.030599      0.034514
    {'Direction_PA:Time_T15'             }          0.011973    0.016618     0.72051    793        0.47143     -0.020647      0.044594
    {'Direction_PA:Current_0'            }           -0.0307     0.01915     -1.6031    793         0.1093     -0.068291     0.0068905
    {'Direction_PA:Current_0.5'          }         -0.039337     0.01915     -2.0541    793       0.040291     -0.076927    -0.0017458
    {'Direction_PA:Current_1'            }        -0.0058802     0.01915    -0.30706    793        0.75888     -0.043471      0.031711
    {'Direction_PA:Current_1.5'          }          0.030124    0.019203      1.5687    793        0.11712    -0.0075714      0.067818
    {'Time_Tbase:Current_0'              }           0.11751    0.033165      3.5433    793     0.00041823      0.052412       0.18261
    {'Time_T00:Current_0'                }         -0.053039    0.033165     -1.5993    793        0.11016      -0.11814      0.012062
    {'Time_T15:Current_0'                }          -0.01957    0.033181    -0.58979    793         0.5555     -0.084703      0.045563
    {'Time_Tbase:Current_0.5'            }         -0.035713    0.033165     -1.0768    793        0.28188      -0.10081      0.029388
    {'Time_T00:Current_0.5'              }          0.036567    0.033165      1.1026    793        0.27055     -0.028535       0.10167
    {'Time_T15:Current_0.5'              }          0.014259    0.033181     0.42973    793        0.66751     -0.050874      0.079392
    {'Time_Tbase:Current_1'              }          -0.10218    0.033165     -3.0808    793      0.0021354      -0.16728     -0.037074
    {'Time_T00:Current_1'                }         0.0014202    0.033165    0.042822    793        0.96585     -0.063681      0.066521
    {'Time_T15:Current_1'                }          0.046986    0.033181       1.416    793        0.15716     -0.018147       0.11212
    {'Time_Tbase:Current_1.5'            }         0.0053859    0.033195     0.16225    793        0.87115     -0.059775      0.070547
    {'Time_T00:Current_1.5'              }         -0.049622    0.033195     -1.4949    793        0.13535      -0.11478      0.015539
    {'Time_T15:Current_1.5'              }          0.034476    0.033456      1.0305    793        0.30309     -0.031197       0.10015
    {'Direction_PA:Time_Tbase:Current_0' }         -0.026503    0.033165    -0.79912    793        0.42446     -0.091604      0.038598
    {'Direction_PA:Time_T00:Current_0'   }          0.018438    0.033165     0.55595    793         0.5784     -0.046663      0.083539
    {'Direction_PA:Time_T15:Current_0'   }          0.004888    0.033181     0.14731    793        0.88292     -0.060245      0.070021
    {'Direction_PA:Time_Tbase:Current_0.5'}         0.022426    0.033165     0.67619    793        0.49912     -0.042675      0.087527
    {'Direction_PA:Time_T00:Current_0.5' }         -0.029223    0.033165    -0.88115    793         0.3785     -0.094324      0.035878
    {'Direction_PA:Time_T15:Current_0.5' }           0.03181    0.033181     0.95868    793        0.33801     -0.033323      0.096943
    {'Direction_PA:Time_Tbase:Current_1' }           0.00307    0.033165    0.092568    793        0.92627     -0.062031      0.068171
    {'Direction_PA:Time_T00:Current_1'   }         -0.011546    0.033165    -0.34814    793        0.72783     -0.076647      0.053555
    {'Direction_PA:Time_T15:Current_1'   }         -0.017177    0.033181    -0.51767    793        0.60483      -0.08231      0.047956
    {'Direction_PA:Time_Tbase:Current_1.5'}        0.0062586    0.033195     0.18854    793         0.8505     -0.058903       0.07142
    {'Direction_PA:Time_T00:Current_1.5' }          0.020529    0.033195     0.61842    793        0.53648     -0.044633       0.08569
    {'Direction_PA:Time_T15:Current_1.5' }          -0.02209    0.033456    -0.66029    793        0.50926     -0.087763      0.043582

Random effects covariance parameters (95% CIs):
Group: Subject (21 Levels)
    Name1                   Name2                   Type          Estimate    Lower       Upper
    {'(Intercept)' }        {'(Intercept)' }        {'std'}        0.40556     0.29654    0.55466
    {'Direction_PA'}        {'Direction_PA'}        {'std'}       0.081897    0.055475     0.1209
    {'Time_Tbase'  }        {'Time_Tbase'  }        {'std'}        0.12229    0.080481    0.18583
    {'Time_T00'    }        {'Time_T00'    }        {'std'}       0.079383    0.045171     0.1395
    {'Time_T15'    }        {'Time_T15'    }        {'std'}       0.055787    0.024094    0.12917
    {'Hour_Morning'}        {'Hour_Morning'}        {'std'}       0.070769    0.013791    0.36317

Group: Subject (21 Levels)
    Name1                Name2                Type           Estimate    Lower      Upper
    {'Session_1'}        {'Session_1'}        {'std'}        0.21926     0.17967    0.26758

Group: Error
    Name               Estimate    Lower      Upper
    {'Res Std'}        0.27746     0.26255    0.29321
```

Notes:

- Categorical predictors were modelled using effects coding.
- Diagonal covariance matrix was used for the first random effect vector and an isotropic covariance matrix for the second random effect vector.

Table S4: Linear mixed effects model of the effects of electric field on the absolute MEP size, related to STAR Methods.

```
Linear mixed-effects model fit by ML

Model information:
    Number of observations            839
    Fixed effects coefficients         38
    Random effects coefficients       210
    Covariance parameters               8

Formula:
    logMEP ~ Session + Gender + Hour + Time*Direction*p1E + Time*Direction*p2E + Time*Direction*p3E ...
        + (1 + Direction + Time + Hour|Subject) + (Session|Subject)

Model fit statistics:
    AIC       BIC       LogLikelihood    Deviance
    698.23    915.91    -303.12          606.23

Fixed effects coefficients (95% CIs):
    Name                                      Estimate      SE          tStat        DF     pValue        Lower        Upper
    {'(Intercept)'                 }             5.8479     0.08998       64.991    801    1.245e-321       5.6713       6.0245
    {'Session_1'                   }           0.090281    0.055102       1.6384    801       0.10172     -0.01788      0.19844
    {'Session_2'                   }          0.0067839    0.055132      0.12305    801        0.9021     -0.10144        0.115
    {'Session_3'                   }           -0.12837    0.054419       -2.359    801      0.018563     -0.23519    -0.021553
    {'Session_4'                   }          0.0066992    0.053725      0.12469    801        0.9008    -0.098759      0.11216
    {'Direction_PA'                }          -0.013865    0.021461     -0.64607    801       0.51842    -0.055991     0.028261
    {'Time_Tbase'                  }           0.016907    0.031791      0.53181    801       0.59501    -0.045497     0.079311
    {'Time_T00'                    }          -0.016221    0.023298     -0.69625    801       0.48648    -0.061954     0.029512
    {'Time_T15'                    }          -0.034373    0.021317      -1.6124    801       0.10726    -0.076217    0.0074713
    {'Hour_Morning'                }          -0.022705    0.036863     -0.61591    801       0.53813    -0.095065     0.049656
    {'Gender_M'                    }           0.072868    0.090008      0.80958    801       0.41842     -0.10381      0.24955
    {'p1E'                         }             1.0423     0.94203       1.1064    801       0.26887     -0.80685       2.8914
    {'p2E'                         }           -0.57719     0.86159     -0.66991    801       0.50311      -2.2684       1.1141
    {'p3E'                         }            0.78799     0.82788      0.95182    801       0.34148     -0.83708       2.4131
    {'Direction_PA:Time_Tbase'     }         -0.0091227    0.016566     -0.55069    801         0.582     -0.04164     0.023395
    {'Direction_PA:Time_T00'       }          0.0019953    0.016566      0.12044    801       0.90416    -0.030522     0.034513
    {'Direction_PA:Time_T15'       }           0.011903    0.016598      0.71716    801       0.47348    -0.020677     0.044483
    {'Direction_PA:p1E'            }            0.96676     0.30133       3.2084    801     0.0013881      0.37528       1.5582
    {'Time_Tbase:p1E'              }           -0.43472     0.51266     -0.84796    801       0.39671       -1.441       0.5716
    {'Time_T00:p1E'                }            0.72193     0.50218       1.4376    801       0.15094     -0.26382       1.7077
    {'Time_T15:p1E'                }           -0.21733     0.49805     -0.43635    801        0.6627       -1.195      0.76031
    {'Direction_PA:p2E'            }          -0.022522     0.29736    -0.075741    801       0.93964     -0.60622      0.56118
    {'Time_Tbase:p2E'              }             1.1739     0.50732        2.314    801      0.020921       0.1781       2.1698
    {'Time_T00:p2E'                }            0.60725      0.4986       1.2179    801       0.22362     -0.37146        1.586
    {'Time_T15:p2E'                }           -0.19637     0.49518     -0.39655    801       0.69181      -1.1684      0.77565
    {'Direction_PA:p3E'            }           0.026405     0.29148     0.090589    801       0.92784     -0.54576      0.59857
    {'Time_Tbase:p3E'              }            -1.5817     0.49973      -3.1652    801     0.0016085      -2.5627      -0.6008
    {'Time_T00:p3E'                }             1.2122     0.49386       2.4546    801      0.014316      0.24282       2.1816
    {'Time_T15:p3E'                }            0.66722     0.49126       1.3582    801       0.17479      -0.2971       1.6315
    {'Direction_PA:Time_Tbase:p1E'}             0.08302      0.4799      0.17299    801        0.8627       -0.859        1.025
    {'Direction_PA:Time_T00:p1E'  }            -0.10337      0.4799      -0.2154    801       0.82951      -1.0454      0.83865
    {'Direction_PA:Time_T15:p1E'  }            -0.41331     0.48046     -0.86025    801       0.38991      -1.3564       0.5298
    {'Direction_PA:Time_Tbase:p2E'}            -0.14901     0.47991     -0.31051    801       0.75625       -1.091      0.79301
    {'Direction_PA:Time_T00:p2E'  }              0.1031     0.47991      0.21484    801       0.82994     -0.83892       1.0451
    {'Direction_PA:Time_T15:p2E'  }            -0.17101     0.48045     -0.35594    801       0.72198      -1.1141      0.77209
    {'Direction_PA:Time_Tbase:p3E'}              0.2365     0.47997      0.49274    801       0.62233     -0.70565       1.1786
    {'Direction_PA:Time_T00:p3E'  }             0.24354     0.47997      0.50741    801       0.61201     -0.69861       1.1857
    {'Direction_PA:Time_T15:p3E'  }            0.053039     0.48016      0.11046    801       0.91207     -0.88949      0.99557

Random effects covariance parameters (95% CIs):
Group: Subject (21 Levels)
    Name1                  Name2                  Type          Estimate    Lower       Upper
    {'(Intercept)' }       {'(Intercept)' }       {'std'}        0.40397     0.29497    0.55324
    {'Direction_PA'}       {'Direction_PA'}       {'std'}       0.088028    0.059376     0.1305
    {'Time_Tbase'  }       {'Time_Tbase'  }       {'std'}        0.12434    0.081849     0.1889
    {'Time_T00'    }       {'Time_T00'    }       {'std'}       0.075074    0.041384    0.13619
    {'Time_T15'    }       {'Time_T15'    }       {'std'}         0.0613    0.028841    0.13029
    {'Hour_Morning'}       {'Hour_Morning'}       {'std'}       0.070569     0.01229    0.40519

Group: Subject (21 Levels)
    Name1               Name2               Type          Estimate    Lower      Upper
    {'Session_1'}       {'Session_1'}       {'std'}       0.22499     0.18442    0.27449

Group: Error
    Name               Estimate    Lower      Upper
    {'Res Std'}        0.27713     0.26224    0.29287
```

Notes:

- Categorical predictors were modelled using effects coding.
- Diagonal covariance matrix was used for the first random effect vector and an isotropic covariance matrix for the second random effect vector.
- The orthonormal polynomials with respect to the observed values of E_{FDI} were:

$$p_1(x) = 0.0745x - 0.0419 \tag{4}$$

$$p_2(x) = 0.1218x^2 - 0.1820x + 0.0377 \tag{5}$$

$$p_3(x) = 0.1950x^3 - 0.5191x^2 + 0.3275x - 0.0294. \tag{6}$$

Table S5: Linear mixed effects model of the effects of time and TMS direction on the MEP latency, related to STAR Methods.

```
Linear mixed-effects model fit by ML

Model information:
    Number of observations            839
    Fixed effects coefficients         35
    Random effects coefficients        84
    Covariance parameters               5

Formula:
    LD ~ Direction*Time + Direction:Current + Time:Current + Direction:Time:Current + (Direction + Time | Subject)

Model fit statistics:
    AIC       BIC       LogLikelihood    Deviance
    898.12    1087.4    -409.06          818.12

Fixed effects coefficients (95% CIs):
    Name                                     Estimate       SE          tStat          DF     pValue        Lower        Upper
    {'Direction_AP'                    }        -0.61905     0.10327        -5.9946    804    3.0784e-09     -0.82175      -0.41634
    {'Time_T00'                        }         -0.1746     0.08601          -2.03    804      0.042682     -0.34343    -0.0057724
    {'Time_T15'                        }        -0.34921    0.092174        -3.7885    804    0.00016284     -0.53014      -0.16828
    {'Time_T30'                        }        -0.44444    0.096464        -4.6073    804    4.7408e-06      -0.6338      -0.25509
    {'Direction_AP:Time_T00'           }       -0.079365     0.13684       -0.57999    804       0.56208     -0.34797       0.18924
    {'Direction_AP:Time_T15'           }        0.047619     0.13684        0.34799    804       0.72794     -0.22098       0.31622
    {'Direction_AP:Time_T30'           }        0.047619     0.13684        0.34799    804       0.72794     -0.22098       0.31622
    {'Direction_AP:Current_0.5'        }       -0.047619     0.11173        -0.4262    804       0.67007     -0.26693       0.17169
    {'Direction_AP:Current_1'          }        -0.14286     0.11173        -1.2786    804        0.2014     -0.36217      0.076456
    {'Direction_AP:Current_1.5'        }       -0.031746     0.11173       -0.28414    804       0.77638     -0.25106       0.18757
    {'Direction_AP:Current_2'          }       -0.047619     0.11173        -0.4262    804       0.67007     -0.26693       0.17169
    {'Time_T00:Current_0.5'            }       -0.063492     0.11173       -0.56827    804       0.57001     -0.28281       0.15582
    {'Time_T15:Current_0.5'            }     -1.6149e-15     0.11173    -1.4454e-14    804             1     -0.21931       0.21931
    {'Time_T30:Current_0.5'            }        0.015873     0.11173        0.14207    804       0.88706     -0.20344       0.23519
    {'Time_T00:Current_1'              }       -0.015873     0.11173       -0.14207    804       0.88706     -0.23519       0.20344
    {'Time_T15:Current_1'              }        0.031746     0.11173        0.28414    804       0.77638     -0.18757       0.25106
    {'Time_T30:Current_1'              }        0.031746     0.11173        0.28414    804       0.77638     -0.18757       0.25106
    {'Time_T00:Current_1.5'            }       -0.047619     0.11173        -0.4262    804       0.67007     -0.26693       0.17169
    {'Time_T15:Current_1.5'            }       -0.039737     0.11325       -0.35088    804       0.72577     -0.26204       0.18256
    {'Time_T30:Current_1.5'            }        0.031746     0.11173        0.28414    804       0.77638     -0.18757       0.25106
    {'Time_T00:Current_2'              }        0.047619     0.11173         0.4262    804       0.67007     -0.17169       0.26693
    {'Time_T15:Current_2'              }        0.047619     0.11173         0.4262    804       0.67007     -0.17169       0.26693
    {'Time_T30:Current_2'              }         0.14286     0.11173         1.2786    804        0.2014    -0.076456       0.36217
    {'Direction_AP:Time_T00:Current_0.5'}       0.063492     0.19352        0.32809    804       0.74293     -0.31637       0.44335
    {'Direction_AP:Time_T15:Current_0.5'}      -0.095238     0.19352       -0.49214    804       0.62276      -0.4751       0.28462
    {'Direction_AP:Time_T30:Current_0.5'}       -0.11111     0.19352       -0.57416    804       0.56602     -0.49097       0.26875
    {'Direction_AP:Time_T00:Current_1'  }        0.14286     0.19352        0.73821    804        0.4606       -0.237       0.52272
    {'Direction_AP:Time_T15:Current_1'  }       0.031746     0.19352        0.16405    804       0.86974     -0.34812       0.41161
    {'Direction_AP:Time_T30:Current_1'  }    -2.5014e-14     0.19352    -1.2926e-13    804             1     -0.37986       0.37986
    {'Direction_AP:Time_T00:Current_1.5'}       0.031746     0.19352        0.16405    804       0.86974     -0.34812       0.41161
    {'Direction_AP:Time_T15:Current_1.5'}      -0.039628      0.1944       -0.20384    804       0.83853     -0.42122       0.34197
    {'Direction_AP:Time_T30:Current_1.5'}      -0.063492     0.19352       -0.32809    804       0.74293     -0.44335       0.31637
    {'Direction_AP:Time_T00:Current_2'  }      -0.015873     0.19352      -0.082023    804       0.93465     -0.39574       0.36399
    {'Direction_AP:Time_T15:Current_2'  }      -0.079365     0.19352       -0.41012    804       0.68183     -0.45923        0.3005
    {'Direction_AP:Time_T30:Current_2'  }       -0.22222     0.19352        -1.1483    804       0.25118     -0.60208       0.15764

Random effects covariance parameters (95% CIs):
Group: Subject (21 Levels)
    Name1                 Name2                   Type          Estimate    Lower       Upper
    {'Direction_AP'}      {'Direction_AP'}        {'std'}       0.30475     0.21858      0.4249
    {'Time_T00'    }      {'Time_T00'    }        {'std'}       0.15582     0.094613     0.25661
    {'Time_T15'    }      {'Time_T15'    }        {'std'}       0.21759     0.14533      0.32577
    {'Time_T30'    }      {'Time_T30'    }        {'std'}       0.25365     0.17405      0.36967

Group: Error
    Name              Estimate    Lower      Upper
    {'Res Std'}       0.36204     0.34409    0.38093
```

Notes:

- The model had no intercept, as the latency difference was zero for the baseline of PA stimulation. For the same reason, there were no fixed effects of Baseline MEP, Session, Current, Hour, or Gender.
- Diagonal covariance matrix was used for the random effects.
- Categorical predictors were modelled using reference coding.
